# Targeted Restoration of the Microbial Tryptophan Oxidative Pathway Ameliorates Autism‐Like Behavioral and Synaptic Deficits

**DOI:** 10.1002/advs.202522265

**Published:** 2026-09-27

**Authors:** Rui Guo, Caian He, Xiao Xiao, Jingmeng Li, Zilong Zhang, Ying He, Chuanchuan Wang, Jiale Zhao, Jun Gong, Jiarui Liang, Tian Yuan, Haiting Sun, Xuebo Liu, Chao Gao, Zhigang Liu

**Affiliations:** ^1^ College of Food Science and Engineering Northwest A&F University Yangling Shaanxi China; ^2^ Northwest A&F University Shenzhen Research Institute Shenzhen Guangdong China; ^3^ College of Chemistry & Pharmacy Northwest A&F University Yangling Shaanxi China; ^4^ Department of Pediatrics Xijing Hospital the Fourth Military Medical University Xi'an China; ^5^ National Institute for Nutrition and Health Chinese Center for Disease Control and Prevention Beijing China; ^6^ Children's Hospital of Fudan University National Children's Medical Center Institute of Nutrition Fudan University Shanghai China; ^7^ Shaanxi Precision Nutrition and Health Research Institute Xi'an Shaanxi China

**Keywords:** autism spectrum disorder, gut‐brain axis, Indole‐3‐acetic acid, microbial tryptophan oxidative pathway, synbiotic intervention

## Abstract

Autism Spectrum Disorder (ASD) is associated with gut microbiota dysbiosis, yet the contribution of specific microbial metabolic pathways remains unclear. Reanalysis of a public ASD cohort revealed reduced functional potential in microbial tryptophan oxidative metabolism. We therefore designed targeted microbial tryptophan oxidative pathway (MTOP) interventions comprising Lacticaseibacillus rhamnosus C502, highland barley β‐glucan, or their synbiotic combination. These interventions enhanced oxidative tryptophan metabolic output and restored associated indole derivatives in ASD fecal cultures. In a VPA‐induced autism‐like mouse model, MTOP‐targeted interventions alleviated behavioral deficits, normalized social‐stimulus‐associated CA3 calcium responses, and improved hippocampal synaptic architecture. These effects were accompanied by gut microbiota remodeling, improved intestinal barrier integrity, increased fecal and brain IAA levels, and restoration of ERK‐CREB‐BDNF‐associated signaling. Microbiota depletion abolished the synbiotic‐mediated benefits, whereas oral IAA supplementation partially reproduced behavioral, neuronal, and selected synaptic improvements. Neuronal AhR knockdown in the retrosplenial cortex attenuated the synbiotic‐mediated rescue, supporting a required role for neuronal AhR. Independent clinical metabolomic analysis supported an association between lower circulating IAA and ASD symptom severity. Collectively, these findings identify impaired MTOP‐related metabolism as a gut‐brain metabolic vulnerability and support MTOP restoration as a candidate synbiotic strategy for ASD‐related conditions characterized by impaired microbial tryptophan oxidative metabolism.

## Graphical abstract

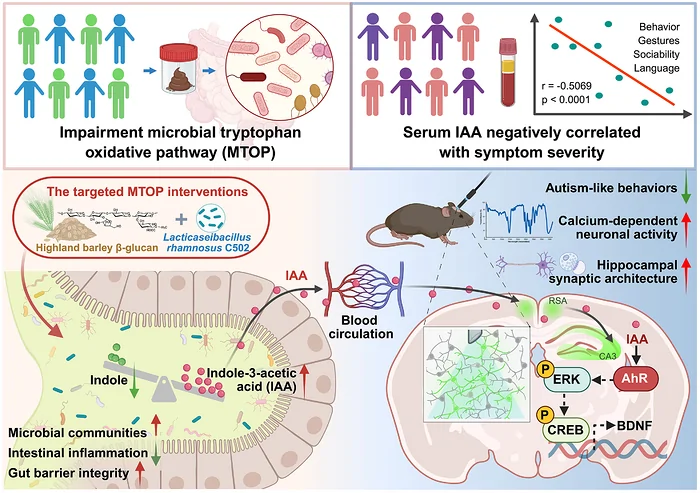

## 1 Introduction

Autism Spectrum Disorder (ASD) is a heterogeneous group of neurodevelopmental disorders characterized by core deficits in social communication, accompanied by restricted and repetitive behaviors and interests [1]. Affecting an estimated 0.6%–1.7% of children globally, with prevalence steadily rising, ASD poses an urgent public health challenge [2]. Although genetic factors contribute substantially to ASD susceptibility, accumulating evidence highlights the importance of environmental influences, particularly during prenatal development. Maternal infection, metabolic dysregulation, and exposure to certain medications have all been identified as risk factors in both clinical and preclinical studies [3, 4]. This period not only represents a sensitive window for neurodevelopment but also coincides with the establishment of the gut microbiota and the maturation of gut‐brain communication [5, 6].

Gastrointestinal disturbances are highly prevalent in individuals with ASD, affecting up to 90% of patients [7], and are increasingly recognized as a clinical hallmark of the disorder. The gut microbiota in ASD patients is profoundly altered, characterized by reduced diversity, compositional imbalance, and decreased production of beneficial metabolites. Although the abundance of certain genera (e.g., Clostridium, *Lactobacillus*, Prevotella, and Bifidobacterium) varies across studies due to differences in diet, geography, or age, the overall pattern consistently reflects microbial dysbiosis [8]. Despite advances in identifying genetic and environmental risk factors, the mechanistic pathways linking microbial disturbances to neurodevelopmental dysfunction remain elusive, and effective therapeutic strategies are still lacking [1].

Recent evidence suggests that gut microbial dysregulation of tryptophan metabolism may represent a crucial mechanistic link between microbiota alterations and ASD neuropathology. In children with ASD, microbial taxa involved in tryptophan catabolism are significantly altered, and their indole‐derived metabolites correlate with both symptom severity and neural activity in specific brain regions [8]. Overall, tryptophan metabolism in ASD appears dysregulated: neuroprotective indole derivatives such as indole‐3‐acetic acid (IAA) are reduced, whereas potentially neurotoxic metabolites, including quinolinic acid and indoxyl sulfate, are increased. This imbalance may promote oxidative stress, neuroinflammation, and mitochondrial dysfunction, thereby exacerbating neural impairment [9, 10]. These findings collectively highlight the microbial tryptophan metabolic network as a promising focal point for understanding ASD pathogenesis.

Microbial metabolism of tryptophan proceeds through three principal routes: a direct cleavage pathway producing indole, an oxidative pathway generating Indole‐3‐acetamide (IAM), indole‐3‐acetaldehyde (IAAld), indole‐3‐acetic acid (IAA), and indole‐3‐aldehyde (IAld), and a reductive pathway yielding indole‐3‐lactic acid (ILA) and indole‐3‐propionic acid (IPA) [11, 12]. In this study, we use MTOP as an operational term to describe the microbial oxidative branch of tryptophan metabolism, involving representative enzymes such as tryptophan decarboxylase (TDC), monoamine oxidase (MAO), IaaDH, TMO, indole‐3‐acetamide hydrolase (IAH), and dye‐decolorizing peroxidases (DyPs). Several of these metabolites, including tryptamine, IAAld, IAA, and IAld, serve as ligands for the aryl hydrocarbon receptor (AhR), a transcription factor that mediates key host responses to microbial metabolites [13, 14, 15]. Activation of AhR signaling exerts anti‐inflammatory, neuroprotective, and barrier‐stabilizing effects, thereby translating microbial metabolic activity into host physiological regulation [16, 17, 18].

Importantly, while tryptophan availability depends on dietary intake, its microbial metabolic fate is strongly influenced by dietary fiber, which determines pathway utilization and metabolite output [19]. Certain fibers can suppress the indole‐producing route and redirect tryptophan flux toward beneficial oxidative metabolites, thereby shaping host physiology through intermicrobial interactions [20, 21]. Thus, targeting the microbial tryptophan metabolic network, particularly through nutritional modulation, offers a promising avenue for influencing gut‐brain axis communication and neurodevelopmental health [22]. Recent metabolomic studies have provided critical clues pointing to dysfunction within a specific segment of this network, the microbial tryptophan oxidative pathway (MTOP). Compared with neurotypical children, those with ASD show reduced urinary levels of oxidative metabolites such as IAA, tryptamine, and IAld, alongside an elevated tryptophan/IAA ratio, with these trends correlating with symptom severity [23]. These findings suggest that MTOP deficiency may represent a mechanistically relevant and therapeutically tractable node within the broader gut‐brain metabolic network.

Building on this insight, we hypothesized that restoring microbial MTOP function could reestablish neurochemical balance and ameliorate autism‐like behavioral and synaptic abnormalities. To test this hypothesis, we developed a targeted MTOP intervention strategy integrating microbial and prebiotic components designed to reinforce the oxidative branch of tryptophan metabolism. By combining multi‐omics analyses, behavioral evaluation, and neurophysiological assays, this study uncovers a previously unrecognized gut‐brain regulatory axis centered on the microbial tryptophan oxidative pathway. Our findings support MTOP restoration as a potential gut‐brain metabolic intervention axis and introduce a synbiotic‐based strategy for ameliorating ASD‐like behavioral and synaptic deficits associated with impaired microbial tryptophan oxidative metabolism.

## 2 Results

### 2.1 Microbial Alterations of Tryptophan Metabolism in Clinically Diagnosed Children With ASD

**FIGURE 1:**
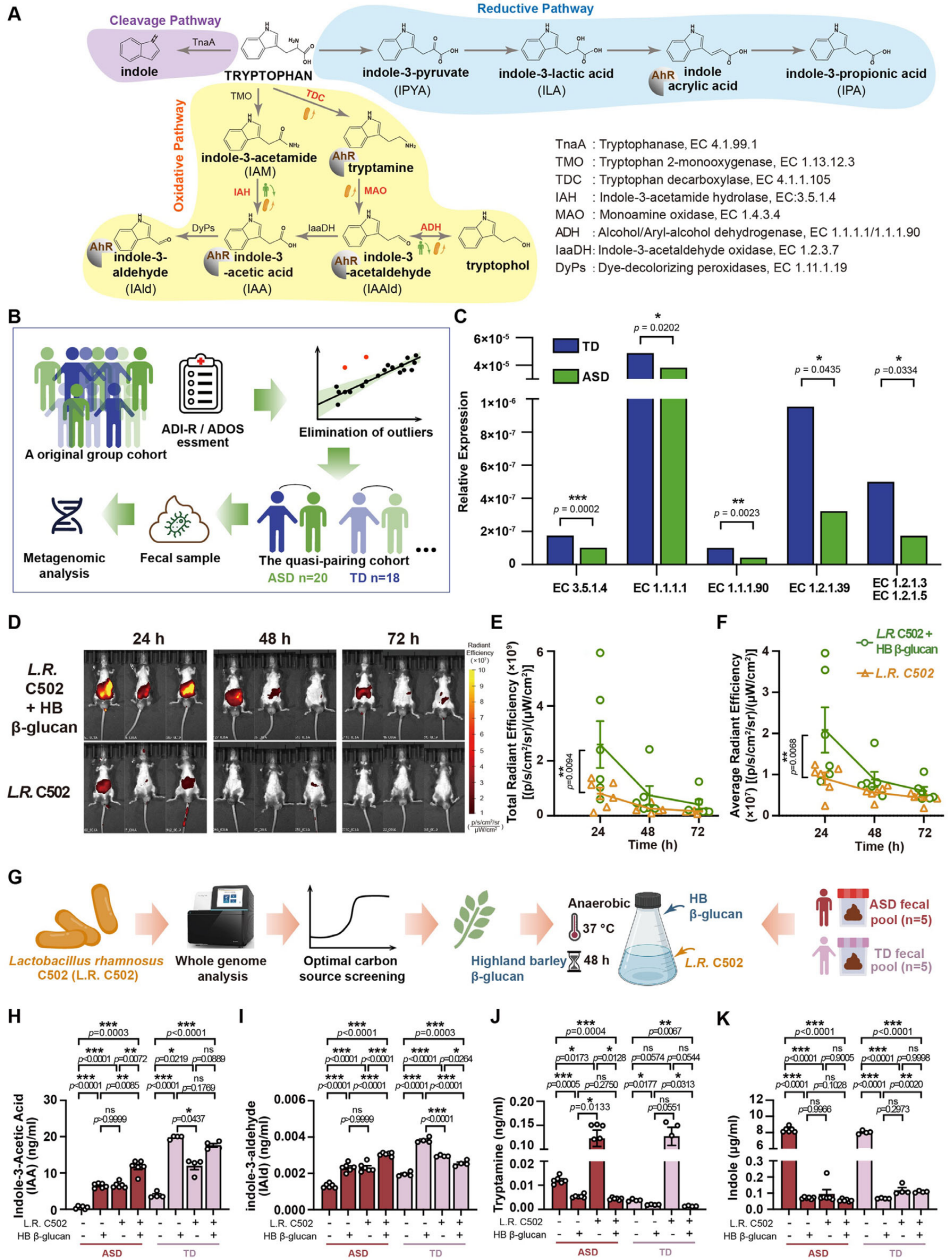
Altered microbial tryptophan metabolism in ASD children and development of a targeted restorative intervention. (A) Schematic of microbial tryptophan metabolic pathways, including the cleavage (purple), reductive (blue), and oxidative (yellow) branches, with key enzymes and metabolites indicated; (B) Workflow of clinical data reanalysis yielding a quasi‐paired cohort of 20 children with ASD and 18 typically developing (TD) controls; (C) Relative expression of genes encoding enzymes in the oxidative pathway of microbial tryptophan metabolism from fecal metagenomic reanalysis, enzymes significantly depleted in ASD are indicated with green human‐shaped icons in (A); Data were obtained from the original study, which applied the Wilcoxon signed‐rank test for paired samples; (D) Representative in vivo imaging of mice at 24, 48, and 72 h post‐gavage with DiR‐labeled *L.R*. C502 alone or the synbiotic (*L.R*. C502 + HB β‐glucan). (E, F) Quantification of total and average Radiant Efficiency (*n* = 6). (G) Formulation and in vitro characterization of the targeted synbiotic strategy; (H–K) Concentrations of IAA, IAld, ILA, tryptamine, and indole in fermentation supernatants after 48 h. ASD pool (*n* = 6 technical replicates) and TD pool (*n* = 4 technical replicates) were generated from five male ASD children and five male TD children, respectively. Data shown as mean ± SEM. Statistical analysis details are provided in Methods.

We reanalyzed stool metagenomes from 20 children with ASD and 18 TD controls to investigate microbial alterations in tryptophan metabolism [24]. As a reference, the principal microbial pathways of tryptophan metabolism and their key enzymes were summarized from the literature (Figure 1A), and gene abundance profiles were extracted from the sequencing dataset (Figure 1B). Analysis revealed a reduced functional potential in selected enzymes of the microbial oxidative branch of tryptophan metabolism in children with ASD. Specifically, genes encoding indole‐3‐acetamide hydrolase (IAH; EC 3.5.1.4), which converts IAM to indole‐3‐acetic acid (IAA), and alcohol/aryl‐alcohol dehydrogenases (ADH; EC 1.1.1.1/1.1.1.90), responsible for interconversion between indole‐3‐acetaldehyde and indole‐3‐ethanol, were significantly downregulated. In addition, genes functionally similar to indole‐3‐acetaldehyde oxidase (IaaDH; EC 1.2.3.7), which oxidizes indole‐3‐acetaldehyde to IAA, were markedly reduced, including those annotated under EC 1.2.1.39/1.2.1.3/1.2.1.5 (Figure 1C). In contrast, genes belonging to other indole‐metabolic branches showed no significant group differences. These findings identify a specific vulnerability of the oxidative pathway in ASD, suggesting a diminished microbial capacity for producing neuroactive tryptophan metabolites.

### 2.2 Targeted MTOP Interventions Reinstated the Oxidative Pathway of Microbial Tryptophan Metabolism

To restore the impaired oxidative pathway, we first selected *Lacticaseibacillus rhamnosus* C502 (*L.R*. C502), a functional microbial strain harboring the key genes IAH, tryptophan decarboxylase (TDC), monoamine oxidase (MAO), and ADH (Figure S1A). To support its efficient growth within the gut, highland barley β‐glucan (HB β‐glucan) was identified as the preferred carbon source promoting robust proliferation (Figure S1B). In vivo colonization was then assessed using whole‐body fluorescence imaging in healthy mice. Unlike the rapid clearance observed after bacterial administration alone, synbiotic intervention (*L.R*. C502 + HB β‐glucan) produced markedly stronger and more persistent intestinal fluorescence over 72 h (Figure 1D–F), indicating improved bacterial persistence. The combination of the functional strain and its specific carbon source was defined as targeted MTOP interventions, comprising three regimens: HB β‐glucan alone, *L.R*. C502 alone, and their synbiotic co‐supplementation.

The metabolic efficacy of these interventions was assessed through in vitro fecal fermentation using samples from children with ASD and TD peers (Figure 1G). HB β‐glucan intervention enhanced production of IAA and IAld (Figure 1H,I), whereas *L.R*. C502 intervention produced similar effects and uniquely increased tryptamine (Figure 1J). Remarkably, the synbiotic intervention exerted synergistic effects in ASD samples, resulting in greater increases in IAA and IAld than either single intervention. Both the single and synbiotic interventions also suppressed indole formation, a cleavage‐pathway product, and reduced L‐tryptophan consumption (Figure 1K, Figure S2A). In addition, synbiotic intervention increased ILA while reducing IPA (Figure S2B,C). Collectively, these results indicate that targeted MTOP interventions reshape microbial tryptophan utilization, with a preferential shift toward the oxidative branch.

### 2.3 Targeted MTOP Interventions Alleviated Social Deficits and Neuronal‐Response Impairment in VPA‐Induced Mice

**FIGURE 2:**
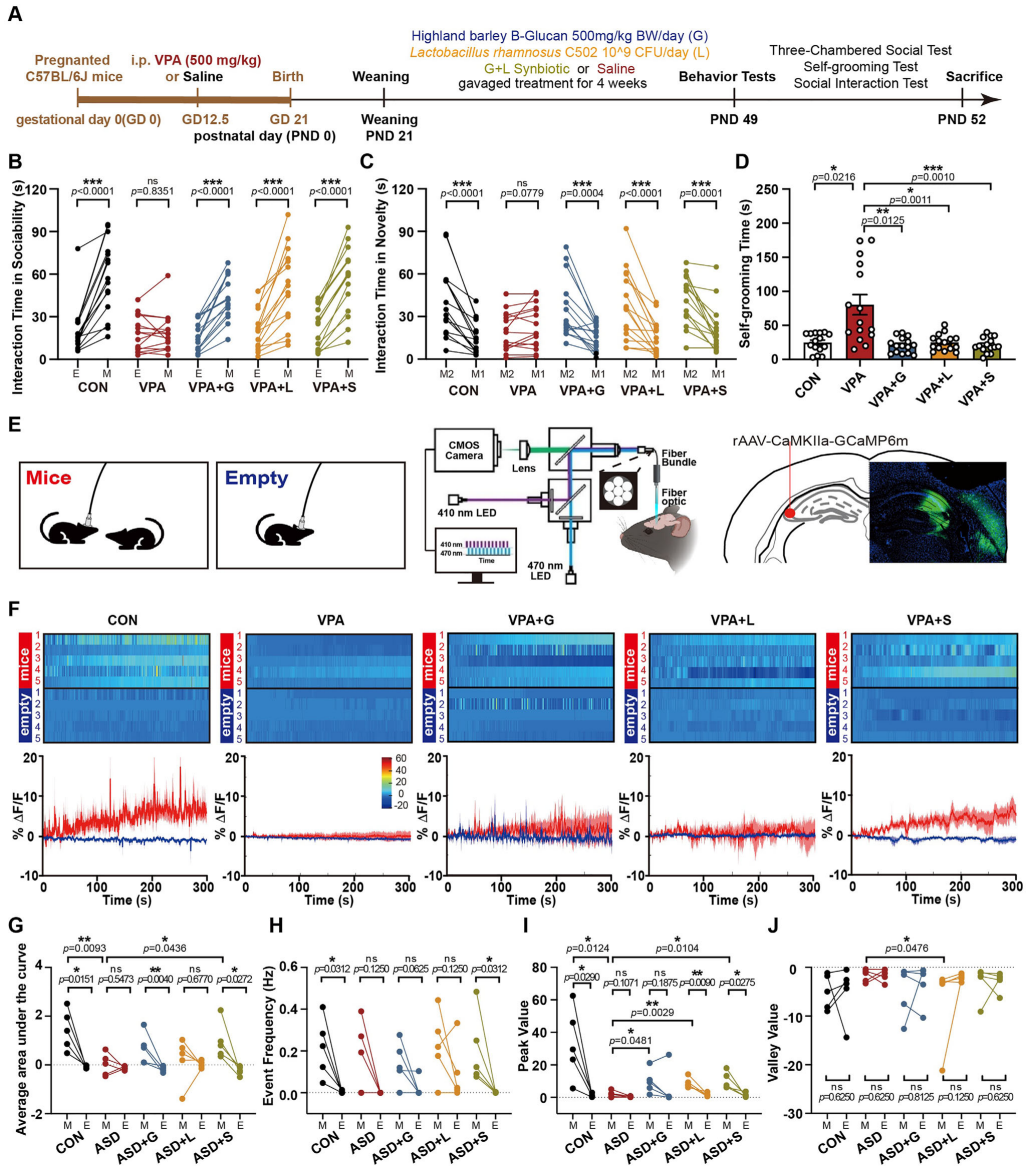
Social behavior and neuronal Ca^2+^ signals in VPA‐induced mice after the targeted intervention strategy. (A) Experimental timeline; (B, C) Interaction time in sociability and social novelty in the three‐chamber test (*n* = 15); (D) Self‐grooming time of mice in the repetitive stereotyped test (*n* = 15); (E) Schematic diagram of fiber photometry calcium signal recording during the social interaction test. (F) Heatmaps (top) and average traces (bottom) of hippocampal CA3 Ca^2^^+^ signals (%ΔF/F) during social interaction (“mice,” red) versus exploration of an empty chamber (“empty,” blue). (G–J) Quantification of GCaMP6m signals including area under the curve, frequency, peak, and valley values (*n* = 5). Data shown as mean ± SEM. Statistical analysis details are provided in Methods.

Male offspring of VPA‐induced dams received targeted MTOP interventions from weaning (3 weeks old) for 4 weeks to restore the microbial oxidative pathway (Figure 2A). Autism‐like social behaviors were evaluated using the three‐chamber test, which measures sociability and preference for social novelty (Figure S3A). Compared with control mice, VPA‐induced mice exhibited no preference for the stranger mouse (M) over the empty cage (E), indicating impaired sociability (Figure 2B). They also failed to distinguish between a familiar mouse (M1) and a novel stranger (M2), reflecting deficits in social novelty (Figure 2C). Targeted MTOP interventions significantly improved both sociability and social novelty performance (Figure S3B,C). Repetitive behaviors were assessed via the self‐grooming test. VPA‐induced mice showed increased grooming time compared to controls, which was markedly reduced after targeted MTOP interventions (Figure 2D). These results demonstrate that targeted MTOP interventions ameliorate autism‐like core social and repetitive behavioral deficits in the VPA model.

Because altered hippocampal excitability contributes to ASD‐related behavioral phenotypes [25], we next examined neuronal responses during social interaction. AAV‐CaMKIIα‐GCaMP6m was injected into the hippocampal CA3 region, and calcium dynamics were recorded in freely moving mice using fiber photometry (Figure 2E). The hippocampal CA3 region was selected because it is a key subregion with defined recurrent circuit architecture and emerging relevance to social behavior and social recognition [26, 27]. In control mice, CA3 neurons displayed significantly higher activity during social than solitary conditions, whereas VPA‐induced mice lacked this difference, indicating impaired responsiveness. Notably, targeted MTOP interventions restored social‐stimulus‐evoked calcium activity in CA3 neurons (Figure 2F). Quantitative analysis revealed that both HB β‐glucan and synbiotic intervention restored significant differences in mean area under the curve between social (M) and non‐social (E) conditions (Figure 2G). Only synbiotic intervention restored social/non‐social differences in event frequency (Figure 2H), while both *L.R*. C502 and synbiotic intervention restored peak amplitude (Figure 2I). HB β‐glucan intervention also increased valley value compared with the VPA group (Figure 2J), although differences among interventions were nonsignificant. Together, these findings indicate that targeted MTOP interventions reversed VPA‐induced impairments in hippocampal neuronal responsiveness associated with social behavior.

### 2.4 Targeted MTOP Interventions Ameliorated Synaptic Abnormalities in VPA‐Induced Mice

**FIGURE 3:**
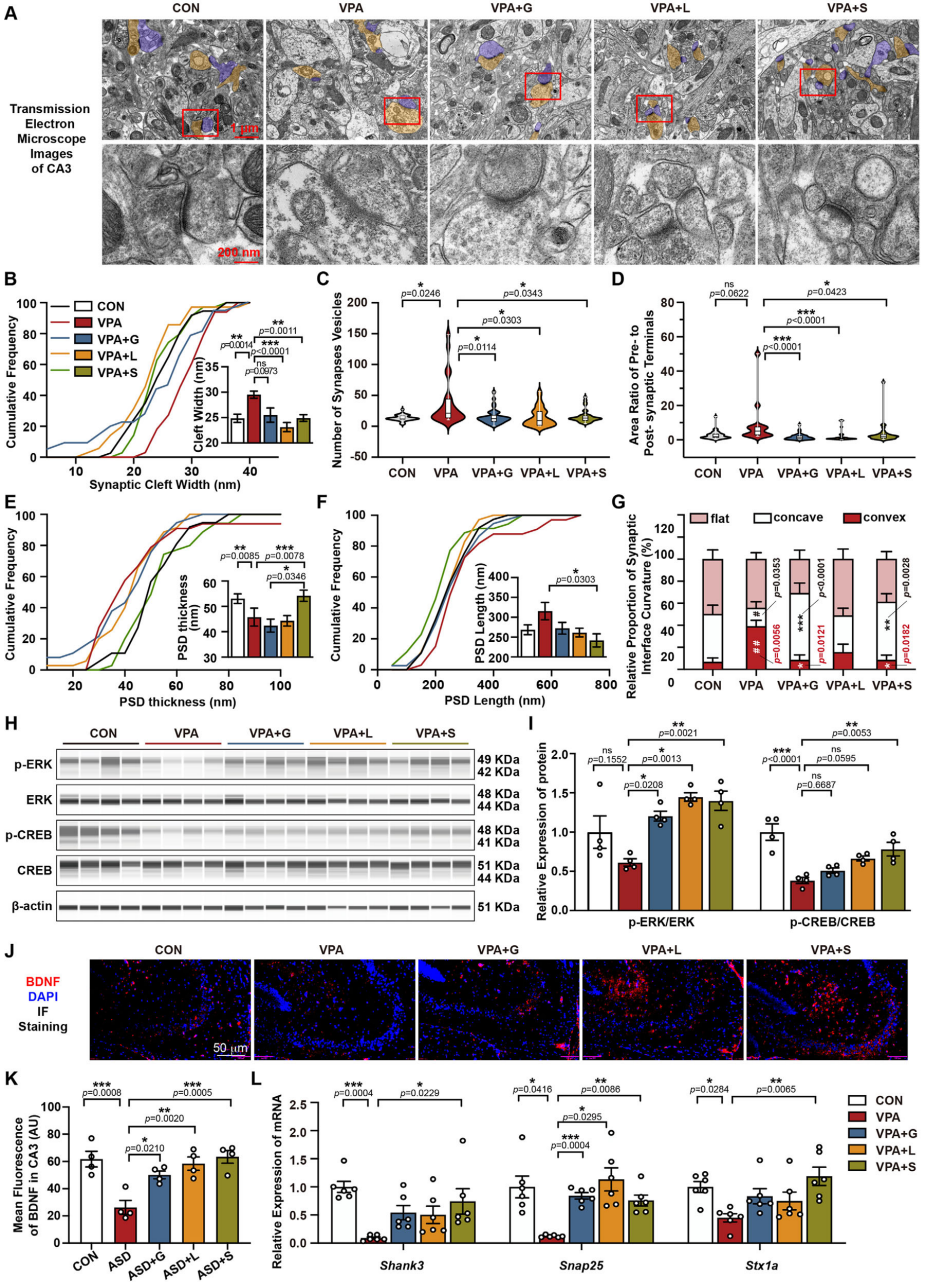
Synaptic signaling and ultrastructure in VPA‐induced mice after the targeted intervention strategy. (A) Representative transmission electron microscopy (TEM) images of CA3 synapses, the presynaptic and postsynaptic terminals are labeled in yellow and purple, respectively; (B–G) Quantification of synaptic cleft width, vesicle number, presynaptic‐to‐postsynaptic area ratio, PSD thickness, PSD length, and synaptic curvature (*n* = 3); (H) Western blots for p‐ERK, ERK, p‐CREB, and CREB in cortex; (I) Quantification of pCREB/CREB and pERK/ERK (*n* = 4); (J‐K) Immunofluorescence images and quantification of BDNF in CA3 region (*n* = 4); (L) Relative mRNA expression of synaptic genes *Shank3*, *Snap25*, and *Stx1a* in the hippocampus (*n* = 6). Data shown as mean ± SEM. Statistical analysis details are provided in Methods.

Given the critical role of structural integrity in neuronal transmission [28, 29], we next examined ultrastructural changes in the hippocampal CA3 region using TEM. In control mice, synapses displayed well‐preserved architecture, with uniform synaptic clefts, evenly distributed vesicles, and intact postsynaptic densities (PSD). In contrast, VPA‐induced mice displayed marked abnormalities, including widened synaptic clefts, blurred PSD, vesicle accumulation, an increased presynaptic‐to‐postsynaptic area ratio, reduced PSD thickness, and altered curvature, characterized by fewer concave and more convex synapses (Figure 3A). Remarkably, targeted MTOP interventions mitigated many of these deficits, normalizing synaptic cleft width (Figure 3B), vesicle number (Figure 3C), and presynaptic‐to‐postsynaptic area ratio (Figure 3D). The synbiotic intervention significantly increased PSD thickness and length (Figure 3E,F), while HB β‐glucan intervention and the synbiotic intervention increased the proportion of concave synapses (Figure 3G). Importantly, H&E and Nissl staining showed no evidence of neuronal loss or gross morphological abnormalities across groups (Figure S4A–C), indicating that structural restoration occurred independently of neuronal degeneration. Together, these results demonstrate that targeted MTOP interventions ameliorate VPA‐induced synaptic abnormalities by promoting hippocampal synapse remodeling and restoring ultrastructural organization.

### 2.5 Targeted MTOP Interventions Restored Molecular Signaling Associated With Synaptic Plasticity

Dysregulation of neurotrophic and transcriptional signaling pathways contributes to ASD pathophysiology [30, 31]. To determine whether targeted MTOP interventions could restore these molecular networks, we examined the ERK‐CREB‐BDNF axis, a key pathway mediating synaptic plasticity. Western blot analysis showed that targeted MTOP interventions significantly increased phosphorylation of extracellular signal‐regulated kinase (ERK) and cAMP response element‐binding protein (CREB) compared with the VPA group (Figure 3H,I), indicating reactivation of the ERK‐CREB signaling axis. Immunofluorescence analysis further demonstrated that brain‐derived neurotrophic factor (BDNF) expression in the hippocampal CA3 region was markedly reduced in VPA‐induced mice but was fully restored by targeted MTOP interventions (Figure 3J,K). In addition, the expression of synaptic scaffolding and vesicle‐associated genes, *Shank3*, *Snap25*, and *Stx1a*, was significantly upregulated following targeted MTOP interventions (Figure 3L). These findings indicate that targeted MTOP interventions reactivate the ERK‐CREB‐BDNF signaling pathway and enhance the transcriptional support required for synaptic functionality, aligning molecular restoration with the observed improvements in synaptic structure and behavior.

### 2.6 Targeted MTOP Interventions Modulated Tryptophan Metabolism in VPA‐Induced Mice

**FIGURE 4:**
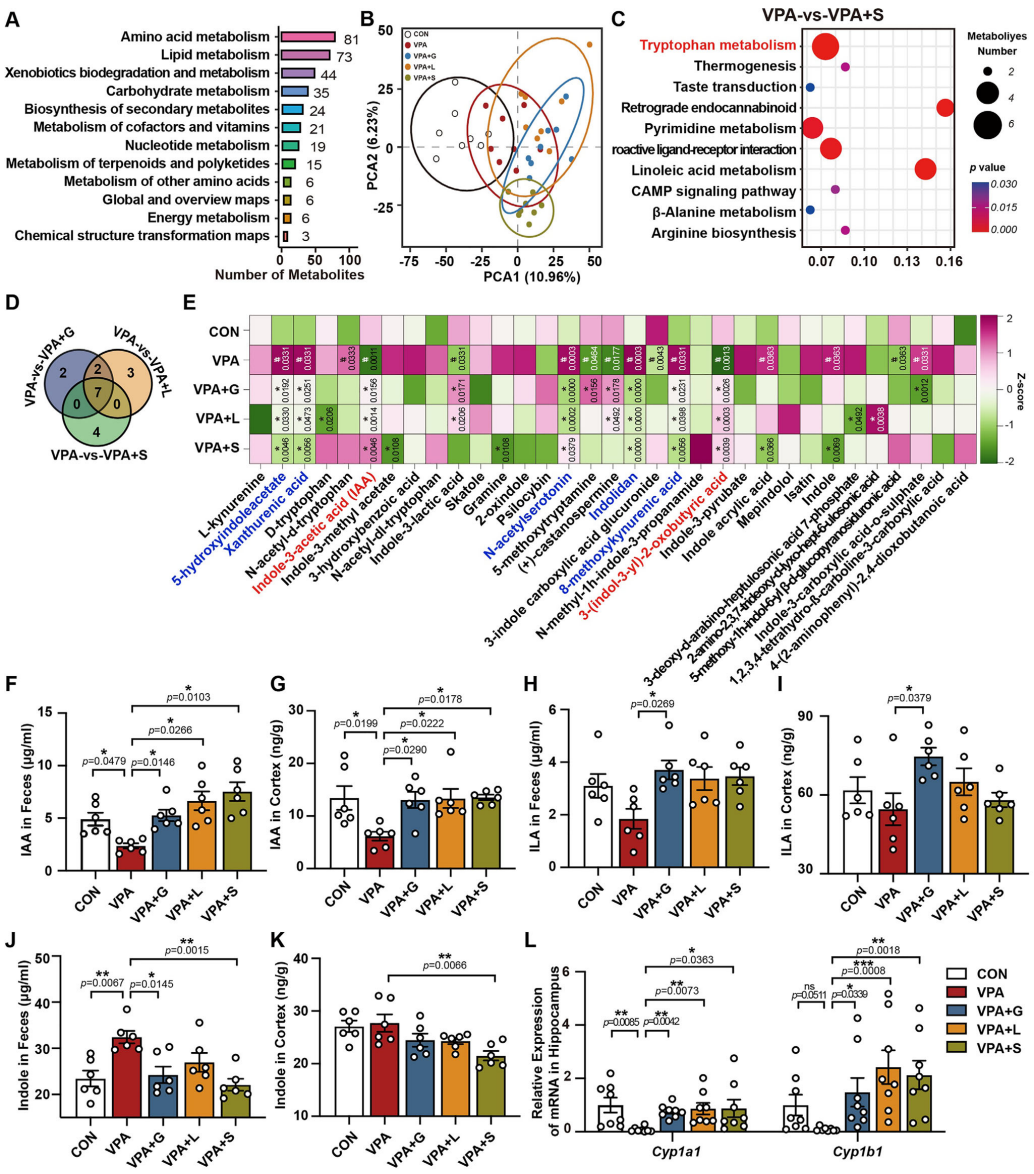
Tryptophan metabolism in VPA‐induced mice after the targeted intervention strategy. (A) KEGG pathway classification of differential serum metabolites (*n* = 8); (B) Principal component analysis of serum metabolomic profiles; (C) KEGG enrichment analysis of differential metabolites between groups (*n* = 8); (D) Venn diagram of tryptophan‐related metabolites; (E) Heatmap of tryptophan‐related metabolites (*n* = 8, numbers indicate q values (Benjamini‐Hochberg FDR‐adjusted p values), and symbols indicate metabolites with *q* < 0.05 and variable importance in projection (VIP) > 1; # denotes VPA versus CON, whereas ^*^ denotes VPA+G, VPA+L, or VPA+S versus VPA); (F–K) Quantification of IAA, ILA, and indole in feces and cortex (*n* = 6); (L) Relative mRNA expression of AhR downstream targets *Cyp1a1* and *Cyp1b1* in hippocampus (*n* = 8). Data shown as mean ± SEM. Statistical analysis details are provided in Methods.

To elucidate the metabolic consequences of MTOP supplementation, we performed untargeted LC‐MS/MS metabolomic profiling of serum samples. A total of 1727 metabolites with annotation confidence levels 1–4 were identified, among which 333 metabolites were mapped to Kyoto Encyclopedia of Genes and Genomes (KEGG) superpathways (Figure 4A). Principal component analysis (PCA) showed a partial separation trend among groups, suggesting that targeted MTOP interventions were associated with changes in the serum metabolic profile (Figure 4B). KEGG enrichment analysis highlighted significant modulation of tryptophan metabolism, particularly among indole derivatives derived from microbial metabolism (Figure 4C, Figure S5A,B).

A focused analysis of tryptophan‐related metabolites from the untargeted metabolomics dataset revealed consistent regulation of eight tryptophan‐related metabolites across all treatment groups, including six downregulated metabolites (5‐hydroxyindoleacetate, xanthurenic acid, N‐acetylserotonin, indole‐3‐carboxylic acid‐O‐sulphate, indolidan, and 8‐methoxykynurenic acid) and two upregulated metabolites (IAA and 3‐(indol‐3‐yl)‐2‐oxobutyric acid) (Figure 4D,E, Table S3). Targeted LC‐MS/MS quantification further confirmed that all interventions significantly increased IAA concentrations in both fecal and brain samples (Figure 4F,G). Additionally, HB β‐glucan intervention significantly increased ILA levels in both fecal and brain tissue (Figure 4H,I), while only the synbiotic intervention effectively reduced indole concentrations (Figure 4J,K). At the molecular level, hippocampal expression of AhR downstream targets, *Cyp1a1* and *Cyp1b1*, was upregulated in all intervention groups, consistent with activation of AhR‐responsive signaling (Figure 4L). Collectively, these findings indicate that MTOP supplementation reprograms microbial tryptophan metabolism in vivo, characterized by enhanced IAA production, reduced indole accumulation, and activation of AhR‐mediated signaling in the brain. The synbiotic formulation showed the most coordinated metabolic and molecular effects among the tested interventions.

### 2.7 Targeted MTOP Interventions Restored Intestinal Barrier Integrity in VPA‐induced Autism‐Like Mice

**FIGURE 5:**
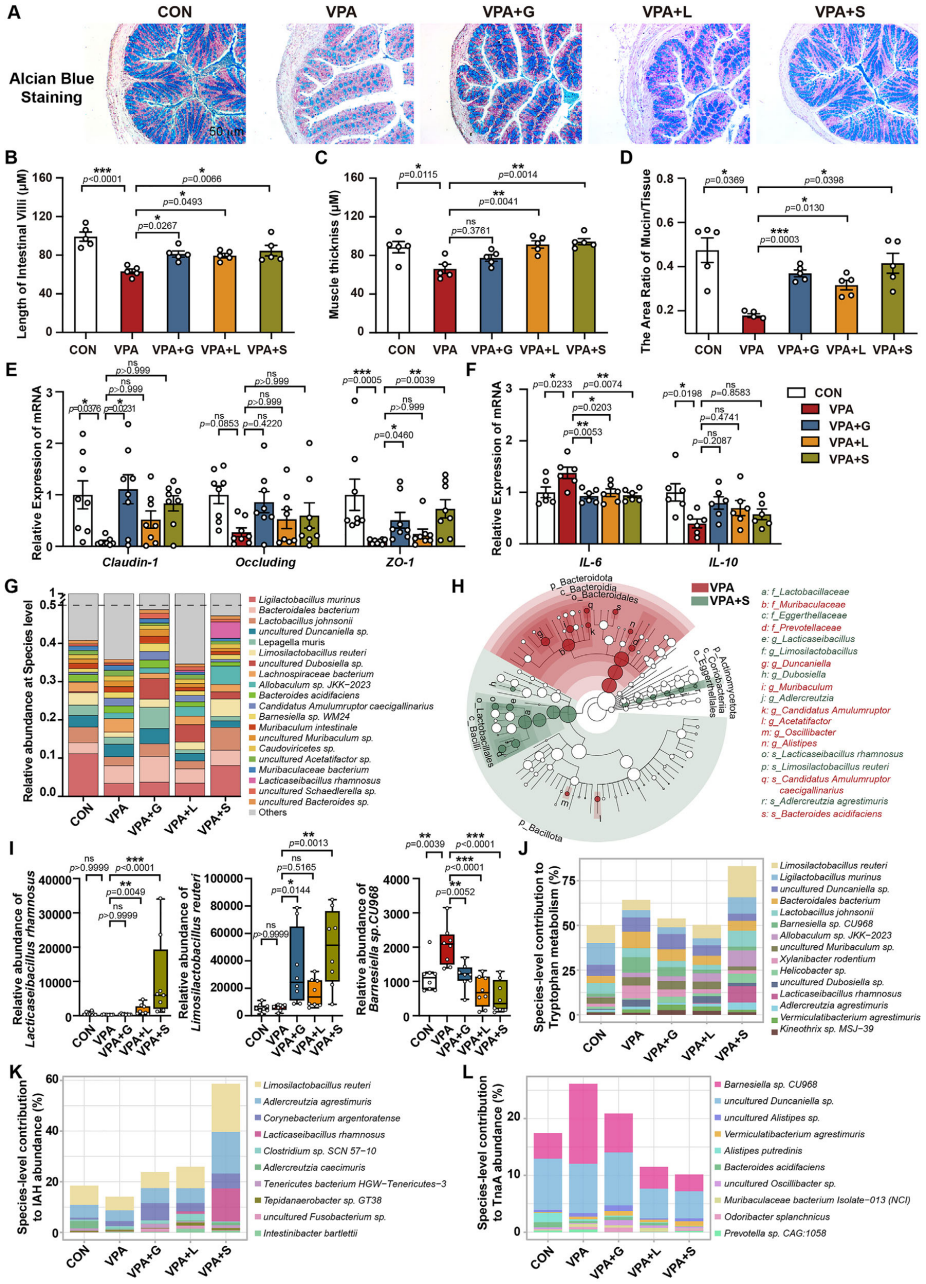
Intestinal barrier function and gut microbiota remodeling after the targeted intervention strategy. (A) Representative images of Alcian Blue staining of colon sections; (B–D) Quantification of colon villi length, muscle thickness, and mucin/tissue ratio (*n* = 5); (E, F) Relative mRNA expression of tight junction proteins (*Claudin‐1, Occludin* and *ZO‐1*, *n* = 8) and inflammatory cytokines (*IL‐6* and *IL‐10*, *n* = 6) in the colon; (G) Relative abundance of gut microbiota at the species level; (H) LEfSe analysis of the VPA and VPA+S groups; (I) Relative abundance of key bacterial strains; (J–L) Species‐level contribution to overall microbial tryptophan metabolism, IAH abundance, and TnaA abundance. Data shown as mean ± SEM. Statistical analysis details are provided in Methods.

Since impaired intestinal permeability contributes to systemic inflammation and neurodevelopmental dysfunction in ASD, we next examined whether targeted MTOP interventions improved gut barrier integrity in VPA‐induced mice. H&E staining and Alcian blue staining were performed to evaluate intestinal barrier integrity (Figure 5A, Figure S6). VPA‐induced mice displayed villi distortion and reduced muscularis thickness, changes that were markedly alleviated by targeted MTOP interventions (Figure 5B,C). Goblet cell numbers, which were decreased in VPA mice, were significantly restored following targeted MTOP interventions (Figure 5D). Tight‐junction integrity was further assessed. The expression of *Claudin‐1* and *ZO‐1*, both key components of the epithelial tight junction complex, was markedly reduced in VPA mice but substantially restored by targeted MTOP interventions. VPA exposure also produced a pro‐inflammatory profile, characterized by increased IL‐6 and decreased IL‐10 levels. Consistent with structural recovery, targeted MTOP interventions normalized IL‐6 expression, indicating attenuation of intestinal inflammation. These findings indicate that MTOP supplementation repairs gut barrier integrity and reduces intestinal inflammation in the VPA model.

### 2.8 Targeted MTOP Interventions Modulated Gut Microbiota Composition in VPA‐Induced Mice

To further resolve species‐level microbial changes and functional contributors underlying MTOP restoration, we performed fecal metagenomic sequencing. Alpha diversity analysis showed that targeted MTOP interventions partially improved microbial diversity, particularly reflected in the Shannon and Simpson indices (Figure S7A). PCA analysis showed only a partial separation trend among groups, with substantial overlap among the VPA‐related intervention groups, suggesting moderate intervention‐associated changes in the overall microbial structure (Figure S7B). Species‐level composition analysis further showed that targeted MTOP interventions altered the relative abundance of multiple dominant species (Figure 5G). LDA analysis identified distinct species‐level microbial signatures associated with different interventions. Compared with the VPA group, the synbiotic intervention was characterized by a discriminative microbial profile involving *Lacticaseibacillus rhamnosus*, *Limosilactobacillus reuteri*, *uncultured Duncaniella sp*., *Adlercreutzia agrestimuris*, and other taxa (Figure 5H). The LDA profiles of HB β‐glucan alone and *L.R*. C502 alone also showed intervention‐specific microbial signatures, including changes in taxa such as *Lepagella muris*, *Dubosiella muris*, *Muribaculum intestinale*, *Mucispirillum schaedleri*, and *Lacticaseibacillus rhamnosus* (Figure S7C,D). Consistent with these species‐level signatures, abundance analysis showed that synbiotic intervention markedly increased *Lacticaseibacillus rhamnosus*. HB β‐glucan and synbiotic interventions increased *Limosilactobacillus reuteri*, whereas all three MTOP interventions reduced *Barnesiella sp*. CU968 (Figure 5I).

To link species‐level remodeling with tryptophan metabolic function, we next analyzed microbial functional contributions to tryptophan metabolism‐related enzymes. The synbiotic intervention showed the highest species‐level contribution to overall microbial tryptophan metabolism (Figure 5J). For indole‐3‐acetamide hydrolase (IAH), which catalyzes the conversion of IAM to IAA, the synbiotic group showed the strongest species‐level functional contribution to IAH‐associated gene abundance, involving multiple taxa, including *Limosilactobacillus reuteri*, *Adlercreutzia agrestimuris*, and *Lacticaseibacillus rhamnosus* (Figure 5K). By contrast, the contribution to TnaA, the tryptophanase responsible for converting tryptophan into indole, was increased in VPA‐induced mice but reduced after targeted MTOP interventions, particularly through decreased contribution from *Barnesiella sp*. CU968 (Figure 5L). Together, these metagenomic results indicate that targeted MTOP interventions, especially the synbiotic combination, reshaped species‐level microbial composition and shifted tryptophan metabolic potential away from the TnaA‐dependent indole‐producing branch and toward IAH‐associated IAA generation.

### 2.9 Metabolic Characteristics of *L.R*. C502

To further verify whether *L.R*. C502 directly contributes to IAA generation through the IAH route, we examined its tryptophan‐derived metabolic profile in vitro. *L.R*. C502 was cultured in MRS medium supplemented with different concentrations of IAM, and metabolite production was quantified by LC‐MS/MS. *L.R*. C502 did not produce detectable IAA in the absence of IAM, whereas IAM supplementation induced a concentration‐dependent increase in IAA production (Figure S8A). Meanwhile, IAM levels remained largely determined by the supplemented substrate concentration and were not increased by *L.R*. C502 itself (Figure S8B). IAld was detected in cultures containing *L.R*. C502, but did not show a clear IAM dose‐dependent pattern (Figure S8C).

We further assessed whether *L.R*. C502 could generate other major tryptophan‐derived metabolites under standard culture conditions. Compared with control medium, *L.R*. C502 did not increase L‐tryptophan consumption or produce detectable increases in indole, tryptamine, ILA, or IPA (Figure S8D–H). These results indicate that *L.R*. C502 is not a broad *de novo* producer of tryptophan‐derived indole metabolites under the tested conditions. Instead, it primarily contributes to IAA generation by converting available IAM to IAA, consistent with its enzymatic profile showing the presence of IAH but absence of TMO. This supports the interpretation that *L.R*. C502 acts as an IAM‐utilizing contributor within the MTOP network.

### 2.10 Correlation Analysis of Behavior, Synaptic Structure, Metabolite, and Gut Microbiota

**FIGURE 6:**
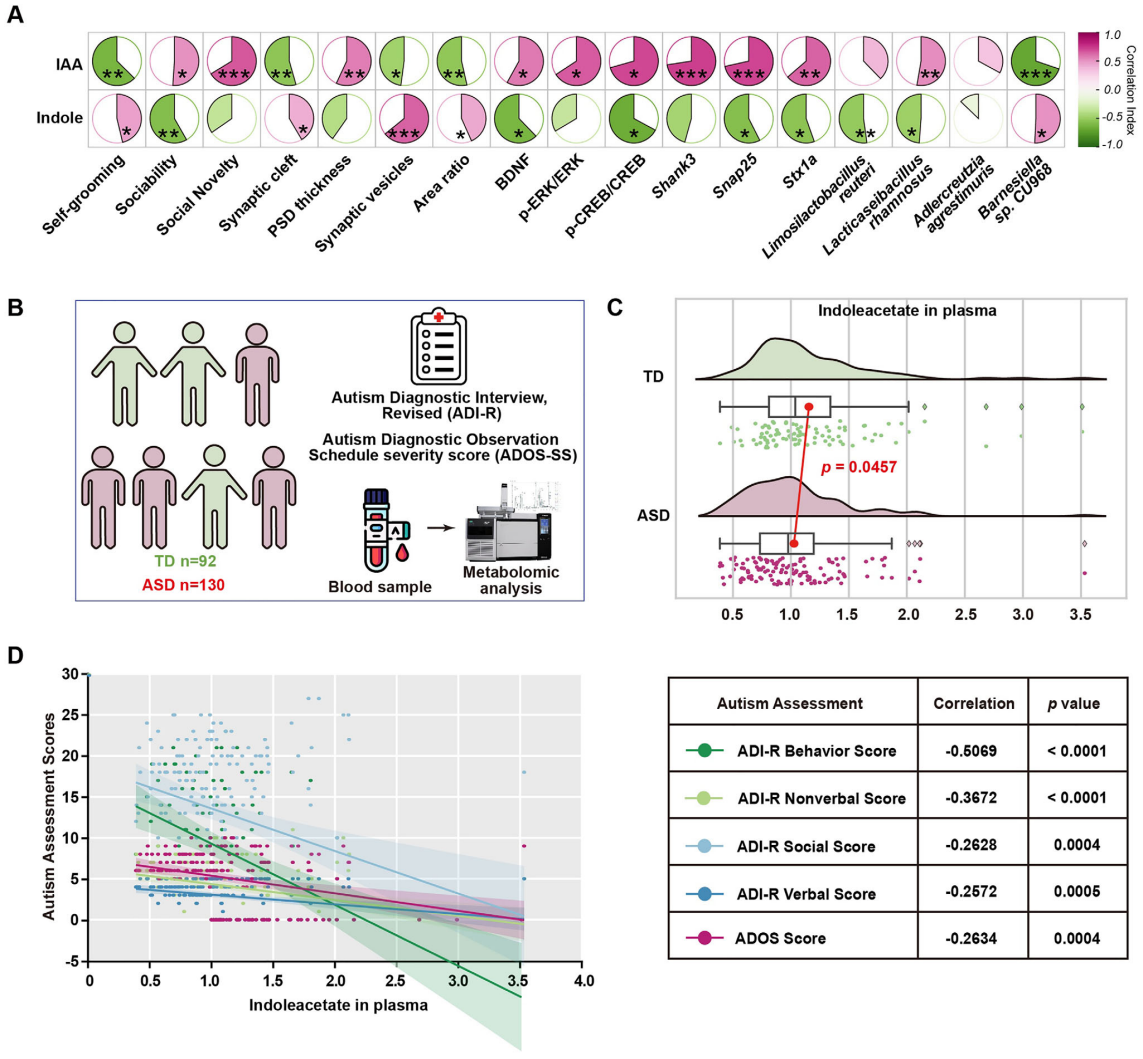
Correlation analysis of gut‐brain associations and clinical relevance. (A) Spearman correlation analysis of key metabolites with behavioral, synaptic, and microbial factors; (B) Schematic of the reanalyzed clinical cohort, including 130 children with ASD and 92 TD controls; (C) Serum indoleacetate levels in ASD and TD children; (D) Spearman correlation of serum indoleacetate levels with ADI‐R and ADOS scores. Data shown as mean ± SEM. Statistical analysis details are provided in Methods.

To elucidate gut‐brain associations, Spearman correlation analysis was performed across behavioral, synaptic, metabolic, and microbial datasets (Figure 6A). This analysis revealed that the microbial oxidative branch of tryptophan metabolism appeared as a central hub linking gut microbes to synaptic and behavioral outcomes. The principal oxidative metabolite, IAA, correlated positively with pro‐social behaviors (sociability, social novelty), synaptic health measures (synaptic thickness and BDNF, *Shank3*, *Snap25*, and *Stx1a* expression), and the abundance of *Lacticaseibacillus rhamnosus*. By contrast, indole, a product of the cleavage pathway, correlated with adverse outcomes, including increased repetitive behaviors and impaired sociability. Additional correlation analysis including other serum tryptophan‐related metabolites further revealed broad associations among tryptophan metabolic remodeling, representative microbial taxa, synaptic parameters, ERK/CREB/BDNF signaling, and behavioral outcomes (Figure S9). Together, these results support the involvement of a broader tryptophan metabolite‐microbiota‐synapse‐behavior network, with IAA representing a key but not exclusive metabolite associated with MTOP restoration.

### 2.11 Independent Clinical Metabolomic Analysis Links Lower Serum IAA to ASD Diagnosis and Symptom Severity

Given the strong association between IAA and autism‐like behaviors observed in our mouse model, we further examined its clinical relevance. Serum metabolomic data from a previously published cohort by Needham et al. [32], comprising children with ASD and age‐matched TD controls, were reanalyzed. ASD was diagnosed using the ADOS and the ADI‐R, based on DSM‐IV criteria, while TD children were screened with the Social Communication Questionnaire (SCQ) and included if their scores were <15 (Figure 6B). The reanalysis revealed that serum IAA levels were significantly lower in children with ASD compared to TD controls (Figure 6C). Furthermore, correlation analysis demonstrated that serum IAA levels were negatively correlated with the ADOS severity scores, including behavioral performance, nonverbal communication, social interaction, and verbal ability (Figure 6D, Table S4). Together with findings from the VPA‐induced autism‐like mouse model, these data provide independent associative support that lower circulating IAA is linked to ASD diagnosis and symptom severity.

### 2.12 Microorganisms Associated With Tryptophan Metabolism Mediate Synbiotic Effects in VPA‐Induced Mice

**FIGURE 7:**
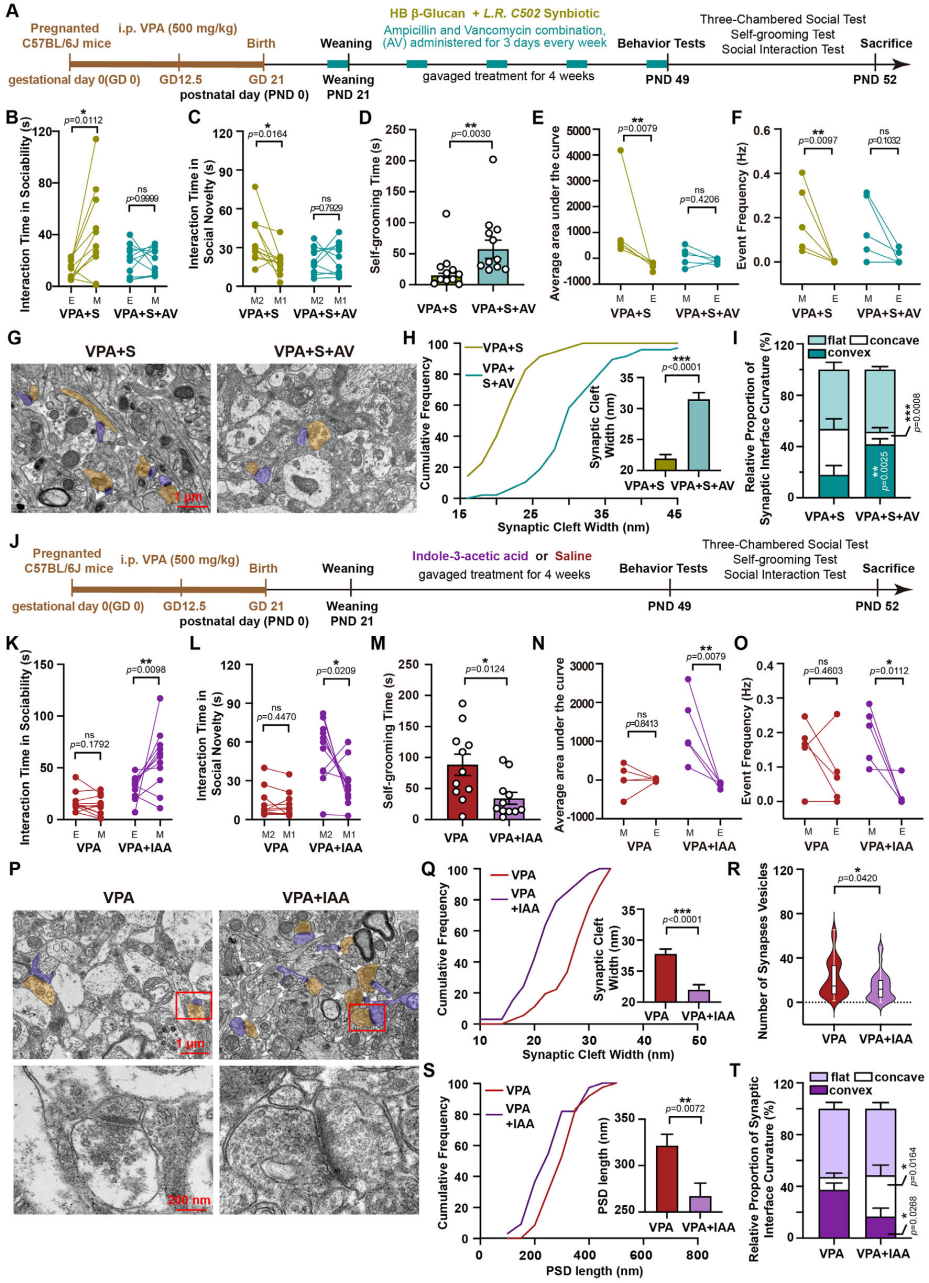
Essential role of microbial tryptophan metabolism and IAA in the targeted intervention strategy‐driven neuroprotection. (A) Antibiotic treatment scheme with ampicillin and vancomycin for depletion of tryptophan‐metabolizing microbiota; (B–D) Behavioral assessments for sociability, social novelty, and self‐grooming (*n* = 11); (E, F) Quantification of GCaMP6m signals including area under the curve, and frequency (*n* = 5); (G) Representative TEM images of CA3 synapses; (H, I) Quantification of synaptic cleft width, and synaptic interface curvature (*n* = 3 for VPA+S group, *n* = 4 for VPA+S+AV group); (J) Experimental design of oral IAA administration; (K–M) Behavioral assessments for sociability, social novelty (n = 11), and self‐grooming (*n* = 10); (N, O) Area under the curve, and event frequency of GCaMP6m signals (*n* = 5); (P) TEM images of CA3 synapses; (Q–T) Quantification of number of synaptic cleft width, synaptic vesicles, PSD length, and synaptic interface curvature (*n* = 3). Data shown as mean ± SEM. Statistical analysis details are provided in Methods.

To determine whether tryptophan‐metabolizing microbiota are required for the MTOP‐mediated effects, indole‐producing bacteria were depleted in VPA‐induced mice using a combination of ampicillin and vancomycin (AV) (Figure 7A) [17, 33]. AV treatment markedly abolished the behavioral improvements observed under targeted MTOP interventions, including enhancements in social interaction, social novelty recognition, and reductions in stereotypic behaviors (Figure 7B–D, Figure S10A,B). Fiber photometry recordings in the hippocampal CA3 region showed that the restoration of neuronal activity induced by targeted MTOP interventions was also eliminated following AV administration (Figure 7E,F), with no significant changes in mean area under the curve, event frequency, or peak amplitude (Figure S10C,D).

Ultrastructural analyses further revealed that AV treatment reversed the synaptic improvements conferred by targeted MTOP interventions (Figure 7G–I). Although changes in PSD thickness and synaptic vesicle accumulation trended toward reversal, these did not reach statistical significance, while presynaptic‐to‐postsynaptic area ratio and PSD length remained unaffected (Figure S10E–H). Consistently, AV treatment reduced serum IAA, IPA, and ILA levels, significantly downregulating hippocampal *Snap25* expression, whereas the expression of *Shank3* and *Stx1a* remained unchanged (Figure S10I,J). These results indicate that a functional tryptophan‐metabolizing microbiota is essential for the therapeutic benefits of targeted MTOP interventions, linking the restoration of the microbial tryptophan metabolic pathway to behavioral, neuronal, and synaptic recovery in VPA‐induced mice.

### 2.13 Oral IAA Supplementation Partially Recapitulates Selected MTOP‐Mediated Benefits

To determine which effects of targeted MTOP interventions could be recapitulated by IAA, a key oxidative pathway metabolite, IAA was orally administered to VPA‐induced mice (Figure 7J). Behavioral assays demonstrated that IAA treatment significantly improved social interaction, while reducing stereotypic behaviors in VPA‐induced mice (Figure 7K–M, Figure S11A,B). Fiber photometry further showed enhanced neuronal responsiveness in the hippocampal CA3 region following IAA treatment, as indicated by higher average area under the curve, event frequency, and peak amplitude in response to social stimuli compared with the empty‐cage condition (Figure 7N,O, Figure S11C,D). TEM further revealed that IAA supplementation ameliorated synaptic ultrastructural abnormalities, including widened synaptic clefts, excessive vesicle accumulation, and reduced PSD length, resulting in improved overall morphology (Figure 7P–T). These effects occurred without changes in the presynaptic‐to‐postsynaptic area ratio or PSD thickness (Figure S11E,F). IAA treatment also increased serum IAA levels (Figure S11G) and significantly upregulated hippocampal *Shank3* expression, while the expression of *Snap25* and *Stx1a* remained unchanged (Figure S11H). Collectively, these results indicate that IAA supplementation partially recapitulates the behavioral, neuronal, and selected synaptic benefits of MTOP restoration, highlighting IAA as a key downstream effector while suggesting that full efficacy requires broader microbial metabolic network interactions.

### 2.14 Neuronal AhR in the Retrosplenial Agranular Cortex is Required for Targeted Synbiotic‐Mediated Behavioral and Synaptic Restoration

**FIGURE 8:**
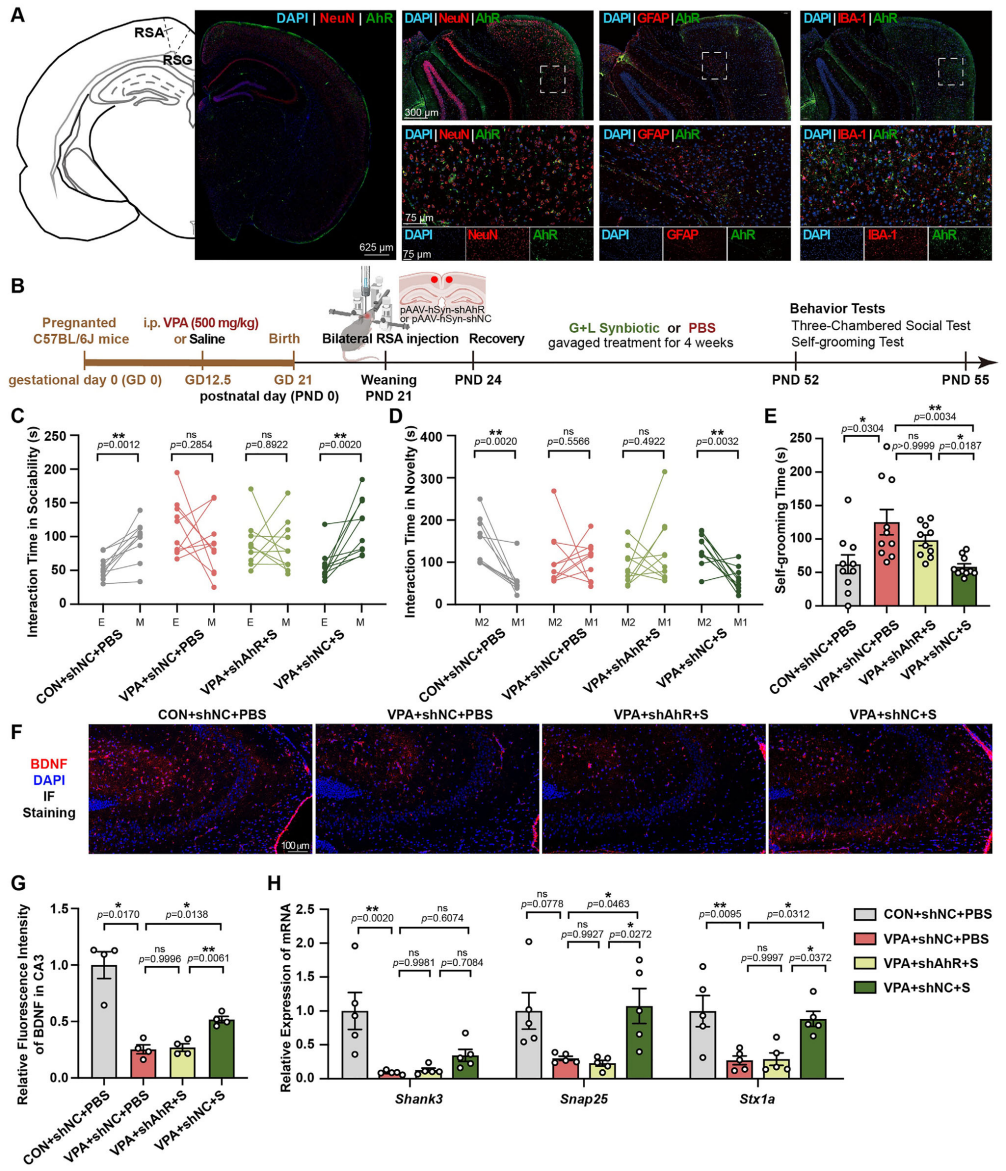
Neuronal AhR in the retrosplenial cortex is required for targeted synbiotic‐mediated neuroprotection. (A) Colocalization of AhR with neurons, astrocytes, or microglia in the RSA region; (B) Experimental design of pAAV‐hSyn‐shAhR bilateral stereotactic injection into the RSA (*n* = 10); (C–D) Interaction time in sociability and social novelty tests (*n* = 10); (E) Self‐grooming time (*n* = 10); (F–G) Representative immunofluorescence images and quantification of BDNF intensity in the CA3 region (*n* = 4); (H) Relative mRNA expression of synaptic genes *Shank3*, *Snap25*, and *Stx1a* in the hippocampus (*n* = 5). Data are shown as mean ± SEM. Statistical analysis details are provided in Methods.

Since IAA is an endogenous ligand of AhR, we further investigated whether neuronal AhR contributes to the synbiotic‐mediated effects of MTOP restoration. We first examined the cellular distribution of AhR in the brain. AhR was prominently expressed in the retrosplenial agranular cortex (RSA) and showed strong colocalization with NeuN‐positive neurons, whereas its colocalization with GFAP‐positive astrocytes or IBA1‐positive microglia was relatively weak (Figure 8A). Retrograde tracing from the CA3 region further labeled neurons in the RSA, supporting an anatomical connection between CA3 and RSA (Figure S12A). Based on these findings, AhR expression was knocked down in RSA neurons by bilateral stereotaxic injection of pAAV‐hSyn‐shAhR, followed by synbiotic intervention during the viral expression period (Figure 8B, Figure S12B).

Behavioral analysis showed that RSA neuronal AhR knockdown abolished the synbiotic‐mediated rescue of sociability and social novelty in VPA‐induced mice, as reflected by both interaction time and interaction index analyses (Figure 8C,D, Figure S12C–E). RSA neuronal AhR knockdown also attenuated the synbiotic‐mediated reduction in self‐grooming behavior (Figure 8E). At the molecular level, synbiotic intervention restored BDNF immunofluorescence in the CA3 region, whereas RSA neuronal AhR knockdown blocked this effect (Figure 8F,G). In addition, RSA neuronal AhR knockdown attenuated the recovery of hippocampal synaptic plasticity‐related genes, particularly Snap25 and Stx1a (Figure 8H). Reduced activation of the canonical AhR target gene Cyp1a1 in cortical tissue after RSA neuronal AhR knockdown further confirmed effective suppression of downstream AhR signaling (Figure S12F,G). Together, these findings indicate that neuronal AhR in the retrosplenial cortex is required for the full behavioral and synaptic restoration mediated by targeted synbiotic intervention.

## 3 Discussion

Altered microbial tryptophan metabolism has been increasingly implicated in the pathophysiology of ASD [8, 11]. In this study, we identified reduced MTOP‐related functional potential in clinically diagnosed children with ASD, including lower abundance of enzymes involved in IAA‐associated oxidative routes and reduced systemic IAA availability. We then developed a targeted MTOP intervention strategy comprising *L.R*. C502, HB β‐glucan, and their synbiotic combination. In VPA‐induced autism‐like mice, these interventions improved social and repetitive behavioral abnormalities, restored social‐stimulus‐associated CA3 neuronal responses, improved hippocampal synaptic ultrastructure, strengthened intestinal barrier integrity, and restored AhR/ERK‐CREB‐BDNF‐associated molecular readouts. The integrated data support a model in which MTOP restoration redistributes microbial tryptophan utilization toward IAA‐associated oxidative metabolism and away from excessive TnaA‐dependent indole generation, while neuronal AhR is required for the synbiotic‐mediated behavioral and synaptic benefits. This framework positions MTOP restoration as a candidate gut‐brain metabolic strategy for ASD‐related conditions characterized by impaired microbial tryptophan oxidative metabolism.

The microbial mechanism of this intervention should be interpreted at the community level. Although *L.R*. C502 was selected to complement the impaired oxidative branch, genomic annotation and substrate‐utilization assays indicate that it is not a complete *de novo* IAA‐producing bacterium. *L.R*. C502 lacks TMO and IaaDH, but converts supplied IAM to IAA in a substrate‐dependent manner, consistent with its IAH‐positive enzymatic profile and supporting its role as an IAM‐utilizing contributor within the MTOP network [34]. In parallel, HB β‐glucan should not be viewed only as a growth‐promoting substrate for *L.R*. C502. It also acts as a fermentable prebiotic that can remodel endogenous microbial members and redirect tryptophan metabolic output, consistent with evidence that dietary fibers shape microbial indole metabolism through community interactions [20, 21]. Metagenomic functional contribution analysis supported this network interpretation. Synbiotic intervention enhanced IAH‐associated contribution involving multiple taxa, including *Lacticaseibacillus rhamnosus*, *Limosilactobacillus reuteri*, and *Adlercreutzia agrestimuris*, while reducing TnaA‐associated contribution, particularly from *Barnesiella sp*. CU968. Accordingly, the synbiotic did not uniformly outperform each single component across every endpoint, but produced the most coordinated profile across IAA‐associated oxidative output, reduced indole generation, microbial functional remodeling, and selected behavioral and synaptic outcomes. The less pronounced in vivo synergy compared with in vitro fermentation likely reflects the greater complexity of the host environment, including host absorption and metabolism, immune‐barrier interactions, ecological niche constraints, microbial competition, and resident microbial cross‐feeding [35, 36]. The upstream microbial sources of IAM and the cross‐feeding interactions remain unresolved and will require further studies.

IAA emerged as a key but partial effector of this network. Previous work has shown that microbial tryptophan metabolites can reach the brain [37], and we detected increased IAA in brain tissue after targeted MTOP interventions. Nevertheless, our data do not determine whether brain IAA reflects direct peripheral transport across the blood‐brain barrier, local metabolism, or indirect gut‐derived signaling. Oral IAA supplementation partially reproduced the behavioral, neuronal, and selected synaptic benefits of MTOP restoration, whereas several ultrastructural and presynaptic gene outcomes required the intact MTOP intervention. These results argue against an IAA‐only model and support a broader microbial metabolic mechanism. Consistent with this interpretation, MTOP‐targeted interventions altered not only IAA but also tryptophan availability and multiple downstream indole derivatives, including indole, IPA, ILA, tryptamine, and IAld. Reduced indole production and increased tryptophan bioavailability may contribute to a more favorable gut‐brain metabolic profile, whereas changes in tryptophan metabolites should be viewed as part of broader metabolic remodeling. Within this framework, neuronal AhR appears to be a required central component of the synbiotic‐mediated effect, as AhR is increasingly recognized as a molecular node linking microbial metabolism with intestinal and neural function [38]. As an endogenous AhR ligand, increased IAA availability may engage neuronal AhR signaling [39, 40]. RSA neuronal AhR knockdown abolished key synbiotic‐mediated behavioral benefits and blocked CA3 BDNF restoration. AhR activation can interact with downstream neuronal regulatory pathways, including the MAPK/ERK pathway, a central regulator of neuronal function [41]. Activation of ERK leads to the phosphorylation of the transcription factor CREB [30], which in turn regulates the expression of synaptic plasticity‐related genes, including BDNF [42]. The accompanying restoration of pERK/ERK, pCREB/CREB, BDNF, and synaptic plasticity‐related genes supports an AhR‐associated ERK‐CREB‐BDNF signaling mechanism, but the precise causal hierarchy from IAA availability to AhR activation and ERK/CREB phosphorylation remains to be experimentally defined.

The synaptic and neurophysiological findings provide a functional context for this metabolic mechanism. VPA‐induced mice showed synaptic cleft widening, PSD disruption, vesicle accumulation, and reduced *Snap25* and *Stx1a* expression, consistent with impaired vesicle cycling and synaptic transmission [43, 44, 45]. Targeted MTOP interventions improved these ultrastructural and molecular defects and increased *Shank3*, a postsynaptic scaffolding gene strongly implicated in ASD‐related synaptic dysfunction [46]. Impaired neuronal responsiveness has been frequently reported in ASD‐related conditions, in which disturbances in excitation‐inhibition balance and calcium signaling may disrupt network synchrony and information processing [47, 48]. CA3 fiber photometry further showed that the VPA model lacked social‐stimulus‐selective neuronal responses, whereas MTOP interventions restored differential CA3 activation during social interaction. In VPA‐induced mice, this blunted CA3 calcium response during social interaction may reflect impaired hippocampal circuit engagement, potentially associated with reduced ERK/CREB signaling and BDNF expression [49, 50]. These calcium dynamics support the relevance of hippocampal functional recovery and are consistent with evidence implicating CA3‐related circuits in social behavior and social recognition [26, 27]. However, CA3 activity should be regarded as one circuit‐level readout rather than the complete neural substrate of the behavioral phenotype; cortical, amygdalar, striatal, and other hippocampal circuits may also contribute to MTOP‐mediated effects.

Peripheral gut mechanisms likely operate in parallel with central neuronal signaling. Targeted MTOP interventions improved intestinal barrier integrity and normalized selected inflammatory markers, including IL‐6 and IL‐10, suggesting that reduced peripheral inflammatory tone may support the gut‐brain effects of microbial tryptophan metabolic remodeling. Importantly, these gut‐brain‐related effects are not necessarily unique to IAA. The overall benefit may arise from coordinated microbial cross‐feeding, redistribution among multiple tryptophan‐derived metabolites, intestinal barrier protection, inflammatory modulation, and neuronal signaling. However, these cytokine measurements do not constitute comprehensive immune profiling, and the relative contribution of barrier repair, immune modulation, and neuronal AhR signaling remains unresolved. Several additional boundaries should temper interpretation. The clinical metagenomic and metabolomic analyses were performed in independent public cohorts that were not fully matched for age, geography, diet, medication history, sampling, or analytical platform; therefore, the human data provide convergent associative evidence rather than definitive clinical validation. The fecal fermentation assay used pooled ASD and TD fecal communities and therefore could not model donor‐level variability. The in vivo work used male offspring in a single VPA‐induced environmental model and assessed outcomes at the end of a 4‐week intervention, leaving sex‐specific effects, durability after withdrawal, and generalizability across genetic, immune, and other environmental ASD‐related models to future studies [51, 52]. Prospective studies combining matched clinical metadata, individualized microbiome‐metabolome profiling, donor‐resolved fermentation, and responder stratification will be needed to define the subgroup applicability of MTOP‐targeted intervention.

In summary, this study identifies impaired microbial tryptophan oxidative metabolism as a gut‐brain metabolic vulnerability associated with ASD‐related phenotypes and demonstrates that MTOP‐targeted intervention can ameliorate autism‐like behavioral and synaptic deficits in a VPA‐induced model. The evidence supports a mechanism involving community‐level tryptophan metabolic redistribution, increased IAA‐associated signaling, neuronal AhR‐dependent contribution, and synaptic restoration, while also indicating that IAA is a key but not exclusive mediator. These findings support further exploration of MTOP restoration as a pathway‐guided synbiotic strategy for metabolically defined ASD‐related subgroups characterized by impaired microbial tryptophan oxidative metabolism and reduced IAA availability.

## 4 Materials and Methods

### 4.1 Clinical Data Reanalyzed

The fecal metagenomic dataset analyzed in this study was obtained from Zhang et al., comprising 39 children with ASD and 40 typically developing (TD) controls aged 3–8 years recruited from the Autism Research Center of Peking University Health Science Center and surrounding communities [24]. The diagnoses of ASD were confirmed with the Autism Diagnostic Interview‐Revised (ADI‐R) and the Autism Diagnostic Observation Schedule (ADOS) according to the *Diagnostic and Statistical Manual of Mental Disorders*, *Fifth Edition* (DSM‐5). To minimize inter‐individual variability, the authors applied a quasi‐paired cohort strategy, in which each ASD sample was computationally matched to a TD sample with a similar metabolic pathway profile in a high‐dimensional feature space. The final quasi‐paired cohort consisted of 20 ASD and 18 TD children, thereby enabling a more robust identification of microbial features specifically associated with ASD. In the original study, microbial species were profiled using MetaPhlAn2, and microbial pathways were annotated using HUMAnN2 according to the BioCyc Database Collection. In the present study, MTOP‐related functional profiles were extracted from the processed functional abundance matrix according to EC/pathway annotations. Because processed functional profiles were used, raw‐sequence annotation parameters such as sequence identity and coverage thresholds were not independently applied in this reanalysis.

The serum dataset utilized in this study was obtained from Needham et al., comprising 130 ASD individuals and 92 TD children aged 3–12 years, convened by the University of California Davis MIND Institute [32]. The diagnosis of ASD was confirmed by professionals using ADOS and ADIR, with higher scores reflecting greater symptom severity. TD participants had Social Communication Questionnaire (SCQ) scores within the normal range, with ADOS and ADIR scores defaulting to 0. We re‐analyzed the serum level of indole acetate in children with ASD and TD, and performed a correlation analysis with ADOS and ADIR scores.

### 4.2 In Vitro Fecal Fermentation Assay

Fecal samples were collected from five male children with ASD and five age‐matched TD males participating in the Clinical Research Protocol on the Relationship Between Gut Microecology and Childhood Psychobehavioral Disorders (GMCPD). The study protocol was approved by the Medical Ethics Committee of the First Affiliated Hospital of Air Force Medical University (approval no. KY20242174‐F‐1), and written informed consent was obtained from parents or legal guardians of all participants. Fecal samples from the five ASD donors and five TD donors were pooled within each group to generate ASD and TD fecal slurries, respectively. Fermentation assays were performed using technical replicates of each pooled fecal community; therefore, inter‐individual variability could not be statistically modeled, and the results were interpreted as pooled‐community metabolic responses rather than donor‐level effects.

Fecal samples were cultured in previously described growth media with minor modifications [53], supplemented with either highland barley β‐glucan (HB β‐glucan, 10 g L^−^^1^, Xi'an Baichuan Biotech Co., Ltd.) or *Lacticaseibacillus rhamnosus* C502 (*L.R*. C502, Guangdong Microbial Culture Collection Center, GDMCC64357), which was originally isolated by our laboratory from traditional homemade yogurt collected in Nagqu, Tibet. Supernatants were collected for metabolite extraction and subsequent analysis as detailed below. All cultures were maintained under anaerobic conditions in a Whitley A95 anaerobic workstation at 37°C, with media pre‐incubated for at least 24 h to ensure hypoxia.

### 4.3 Analysis of Indole Derivatives

The concentrations of tryptophan‐derived metabolites, including IAA, IAM, IAld, indole, tryptamine, ILA, and IPA, were determined using LC‐MS/MS in fecal samples, serum, brain tissues, fermentation supernatants, and bacterial culture supernatants where applicable. For brain tissue samples, weighed cortical tissues were homogenized in pre‐chilled methanol, followed by incubation, centrifugation, and collection of supernatants for LC‐MS/MS analysis. Metabolite levels were normalized to tissue weight. For serum analysis, 30 µL of sample was mixed with 870 µL pre‐chilled methanol to precipitate proteins, incubated at −20°C for 20 min, and centrifuged at 13,000 rpm for 10 min at 4°C. For fecal samples, 1 mL of methanol was added, vortexed for 1 min, incubated in a 40°C water bath for 20 min with intermittent mixing, cooled at −20°C for 20 min, and centrifuged under the same conditions. For the in vitro metabolic assay, *L.R*. C502 was cultured in standard MRS medium supplemented with L‐tryptophan and different concentrations of IAM. After incubation, culture supernatants were collected and processed using the same methanol extraction procedure. Metabolite levels in bacterial culture samples were normalized to bacterial abundance and expressed per 10^8^ CFU. Chromatographic separation was performed on a ZORBAX XDB C18 column (4.6 × 250 mm, 5 µm, 30°C). The mobile phases consisted of 15 mM sodium dihydrogen phosphate (pH 2.8) and methanol, with a flow rate of 1.0 mL/min. The gradient elution program was as follows: 0–12 min (A: B = 42:58), 12–28 min (A: B = 50:50), and 28–35 min (A: B = 85:15). Mass spectrometric detection was performed in multiple reaction monitoring (MRM) mode using electrospray ionization, with compound‐specific ion transitions optimized using authentic standards.

### 4.4 Animals and Drug Treatment

Ten‐week‐old male and female C57BL/6J mice (Beijing HFK Biotechnology Co., Ltd.) were housed under standard laboratory conditions. All animal procedures were approved by the Animal Ethics Committee of Northwest A&F University (approval no. XN2023‐0612). Mating and embryo identification were performed as previously described [54, 55]. On embryonic day 12.5, pregnant dams received a single intraperitoneal injection of saline solution or valproic acid (VPA, 500 mg/kg, i.p., Glpbio, GC11424) [4]. Only male offspring were used for subsequent experiments and were weaned at postnatal day 21.

The first batch of male offspring was randomly divided into five groups, with each group containing mice derived from at least three different dams: (1) CON: prenatal saline exposure with post‐weaning PBS supplementation for 4 weeks; (2) VPA: prenatal VPA exposure with post‐weaning PBS supplementation for 4 weeks; (3) VPA+G: prenatal VPA exposure with post‐weaning supplementation with HB β‐glucan (500 mg/kg BW) for 4 weeks; (4) VPA+L: prenatal VPA exposure with post‐weaning supplementation with *L.R*. C502 (∼1 × 10^8^ CFU/mouse/day) for 4 weeks; (5) VPA+S: prenatal VPA exposure with post‐weaning supplementation with both HB β‐glucan (500 mg/kg BW) and *L.R*. C502 (∼1 × 10^8^ CFU/mouse/day) for 4 weeks.

The second batch of male offspring was randomly divided into two groups: VPA+S and VPA+S+AV. In the VPA+S+AV group, mice were treated with a mixture of ampicillin (500 mg/kg BW) and vancomycin (250 mg/kg BW) by oral gavage for 10 days prior to the synbiotic intervention to deplete tryptophan‐metabolizing gut microbiota [17, 33].

The third batch was assigned to VPA and VPA+IAA groups. In the VPA+IAA group, mice were prenatally exposed to VPA and supplemented with IAA (20 mg/kg BW, Glpbio, GC43901) for 4 weeks post‐weaning. All mice were weighed weekly, subjected to behavioral testing at 8 weeks of age, and subsequently euthanized for collection of feces, serum, and tissue samples.

The fourth batch of male offspring was used for neuronal AhR knockdown experiments. VPA‐induced mice received bilateral stereotaxic injection of pAAV‐hSyn‐shAhR or the corresponding control virus pAAV‐hSyn‐shNC into the retrosplenial agranular cortex (RSA) at postnatal day 21. After viral injection, mice were allowed to recover for 3 days and were subsequently administered synbiotic supplementation or PBS for 4 weeks. The experimental groups were CON+shNC+PBS, VPA+shNC+PBS, VPA+shAhR+S, and VPA+shNC+S. Behavioral testing was performed after the intervention period, followed by tissue collection for immunofluorescence and gene expression analyses.

### 4.5 Behavioral Experiments

Behavioral tests, including the three‐chamber social interaction test and self‐grooming analysis, were conducted to assess sociability, social novelty preference, and repetitive behaviors [54, 55]. For the three‐chamber test, mice were acclimated in a three‐chamber apparatus for 10 min, then allowed to explore either an empty cage (E) in the left chamber or a cage containing an unfamiliar mouse (M1) in the right chamber. Afterward, the empty cage was replaced with a second, novel mouse (M2), and the time spent interacting with each cage was recorded using SuperMaze software. For self‐grooming analysis, mice were acclimated in a clean cage for 10 min, after which grooming behaviors such as paw licking, body grooming, or scratching were observed and recorded.

### 4.6 Fiber Photometry Recording

To investigate how synaptic activity in the brain was modulated by experimental interventions, real‐time calcium dynamics in hippocampal CA3 neurons were monitored using fiber photometry during social interactions. Male mice prenatally exposed to saline or VPA were injected with rAAV‐CaMKIIa‐GCaMP6m (BrainVTA, PT‐0111) into the hippocampus CA3 region (AP, −1.82 mm; ML, ±2.66 mm; DV, −2.54 mm). An optical fiber (ThinkerTech, Nanjing, China) was implanted at the same site.

After a 1‐week recovery period, mice were administered the respective interventions by gavage for 4 weeks. Recordings were conducted using a fiber photometry system (ThinkerTech, Nanjing, China) equipped with 470 and 405 nm LEDs to excite GCaMP6m fluorescence and isosbestic signals, respectively. Excitation light was delivered through a dichroic mirror and a ×10 objective lens into a multimode optical fiber (230 µm O.D., NA = 0.37, 2 m). Laser power was maintained at 40 µW to minimize photobleaching. Fluorescence signals were collected through the same fiber, filtered, demodulated, and digitized at 100 Hz.

Mice were freely moving in an arena during 10 min recordings sessions. On test days, each mouse was first placed in an empty cage for 5 min of exploration, followed by the introduction of an unfamiliar conspecific for 5 min of social interaction. During this period, neuronal activity changes in the CA3 region were recorded under the social behavior paradigm. Fluorescence changes (ΔF/F) were calculated as (F‐F0)/F0, and the normalized Z‐score and areas under the curve (AUC) were derived. Calcium signal peaks and frequencies during social interactions were analyzed in MATLAB. Baseline fluorescence was defined as the mean of ten low‐nadir points, and signals exceeding baseline were extracted for quantitative analysis.

### 4.7 Histopathological Analysis

Histopathological analyses, including hematoxylin and eosin (H&E), Nissl, and Alcian Blue staining, were performed to assess structural alterations in the brain and colon. Brain and colon tissues were collected after euthanasia, fixed in 4% paraformaldehyde, dehydrated, and embedded in paraffin. Sections (5 µm) were mounted on APTS‐coated slides and dried at 37°C. Brain and colon sections were stained with H&E, Nissl solution, or Alcian Blue/nuclear fast red, then cleared and mounted for microscopic examination.

### 4.8 Transmission Electron Microscopy of Brain Samples

Transmission electron microscopy (TEM) was performed to evaluate the ultrastructural synaptic changes in the CA3 hippocampal region across experimental groups [56, 57]. Brain tissues were fixed in ice‐cold fixative for 20 h, followed by post‐fixation in 1% OsO_4_ containing 0.8% K_4_(Fe(CN)_6_). After graded ethanol dehydration, samples were embedded in Epon 812 resin. Ultra‐thin sections (60 nm) were examined using a transmission electron microscope (JEM‐1200EX, Jeol, Japan). Images were captured by a MORADA camera and analyzed using iTEM 1233 software. Synaptic vesicle density was quantified from micrographs acquired at 50 000× magnification, with 30 nerve terminals evaluated per animal. For synaptic ultrastructural quantification, three or four mice were selected from each group. For each mouse, 10–12 synaptic profiles were randomly selected and analyzed. The measured parameters included synaptic cleft width, postsynaptic density length and thickness, presynaptic vesicle number, pre‐/postsynaptic area ratio, and synaptic morphology, which was classified as concave, convex, or flat.

### 4.9 Intestinal Colonization of *L.R*. C502

To verify the intestinal colonization of orally administered *L.R*. C502, in vivo fluorescence tracking was performed following bacterial gavage. Bacterial cells were resuspended in PBS at 1 × 10^8^ CFU/mL and incubated with DiR fluorescent dye (1 mM, 22070, Beijing Fluorescence Biotechnology Co., Ltd.) at 37°C in the dark for 20 min [58]. The suspension was centrifuged (13 000 × g, 10 min, 4°C) and washed three times with sterile PBS to remove unbound dye. The labeled bacteria were re‐suspended in PBS to a final concentration of 1 × 10^9^ CFU/mL, and 0.1 mL was administered to C57BL/6J mice by gavage. Fluorescence imaging was performed on anesthetized mice at 24, 48, and 72 h post‐gavage using an IVIS Lumina III Smart Imaging System (PerkinElmer, USA) with excitation at 748 nm and emission at 780 nm [59].

### 4.10 Western Blot

Protein expression in mouse cortical tissues was analyzed using the automated capillary‐based Jess Western blot system (ProteinSimple, USA), with four mice included in each group. Total proteins were extracted from cortical samples using a protein extraction kit (PL001, ZhongHuiHeCai Biotechnology, China), and protein concentrations were determined with a BCA Protein Assay Kit (A55864, Thermo Fisher Scientific Inc., USA). Before loading, protein samples were diluted with sample buffer, mixed with master mix, and denatured at 95°C for 5 min. The denatured protein samples, chemiluminescent substrate, primary antibodies, horseradish peroxidase‐conjugated secondary antibody, and a biotinylated protein ladder ranging from 12 to 230 kDa were loaded into the designated wells of the assay plate according to the manufacturer's instructions. Automated electrophoretic separation, immobilization, blocking, antibody incubation, washing, and chemiluminescent detection were then performed on the Jess system. Signal acquisition and quantitative analysis were conducted using Compass software (ProteinSimple, USA). The following primary antibodies were used: rabbit anti‐β‐actin (1:50; Abcam, ab8227), rabbit anti‐CREB (1:50; SA04‐04, HUABIO, China), rabbit anti‐phospho‐CREB (Ser133) (1:50; S133, HUABIO, China), mouse anti‐ERK1/2 (1:50; Santa Cruz Biotechnology, sc‐514302), and mouse anti‐phospho‐ERK (1:50; Santa Cruz Biotechnology, sc‐7383).

### 4.11 Immunofluorescence Staining

Immunofluorescence staining was performed to assess regional BDNF expression and the cellular localization of AhR in brain tissue. Paraffin‐embedded brain sections were deparaffinized, rehydrated, and rinsed in PBS. After permeabilization with 0.5% Triton X‐100 and antigen retrieval in sodium citrate buffer at 100°C, endogenous peroxidase activity was quenched with 3% H_2_O_2_. Sections were blocked with goat serum to minimize nonspecific binding.

For BDNF staining, sections were incubated overnight at 4°C with anti‐BDNF primary antibody (1:500; Abcam, UK). After washing, sections were incubated with the corresponding Alexa Fluor‐conjugated secondary antibody (Abways, China) for 2 h at 25°C, counterstained with DAPI, and mounted using antifade medium.

For AhR cellular localization, double immunofluorescence staining was performed to examine the colocalization of AhR with NeuN, IBA1, or GFAP. Sections were first incubated overnight at 4°C with anti‐AhR primary antibody (1:200; sc‐133088; Santa Cruz Biotechnology, USA), followed by incubation with the corresponding Alexa Fluor‐conjugated secondary antibody for 2 h at 25°C. The sections were then incubated overnight at 4°C with one of the following cell marker antibodies: anti‐NeuN (1:500; ab104224; Abcam, UK), anti‐IBA1 (1:500; ab178847; Abcam, UK), or anti‐GFAP (1:500; ab7260; Abcam, UK). On the third day, sections were incubated with the corresponding Alexa Fluor‐conjugated secondary antibody for 2 h at 25°C, counterstained with DAPI, and mounted using antifade medium.

Fluorescent images for Figure 8 and Figure S12 were acquired using a digital pathology fluorescence scanner KF‐FL‐020 (Ningbo Jiangfeng Bio‐information Technology Co., Ltd., China). Other fluorescence images were visualized using an Olympus fluorescence microscope (Tokyo, Japan). Regional fluorescence intensity and colocalization signals were quantified using ImageJ software where applicable.

### 4.12 Quantitative Real‐Time PCR (qRT‐PCR) Analysis

A qRT‐PCR analysis was conducted to measure gene expression in brain, colon, and liver tissues. Total RNA was extracted using BIOZOL reagent (Bioer, Zhejiang, China), and RNA concentration and purity were determined with a NanoDrop 2000 spectrophotometer (Thermo Scientific, MA, USA). cDNA was synthesized from total RNA using UEIris RT mix with DNase (Hengyu Biotech, Jiangsu, China), followed by five‐fold dilution prior to amplification. qPCR reactions were performed using 2× SYBR Green qPCR Master Mix (Hengyu Biotech, Jiangsu, China) under standard cycling conditions. Glyceraldehyde 3‐phosphate dehydrogenase (GAPDH) served as the internal control. Relative gene expression was calculated using the 2^−△△Ct^ method. For brain‐related analyses, hippocampal tissues were used for synaptic plasticity‐related genes, including *Shank3*, *Snap25*, and *Stx1a*, whereas cortical tissues containing the RSA region were used for AhR target genes, including *Cyp1a1* and *Cyp1b1*, in the neuronal AhR knockdown experiment. Primer sequences are listed in Table S1.

### 4.13 Non‐Targeted Metabolomics Assay

To characterize systemic metabolic changes induced by the experimental interventions, non‐targeted metabolomics analysis was conducted on serum samples. Metabolite profiling was performed using an LC‐MS QTRAP 6500+ system (SCIEX) equipped with a BEH C18 column (2.1 mm × 100 mm, 1.7 µm; Waters). High‐resolution analysis was carried out on a UPLC system coupled to a time‐of‐flight mass spectrometer. For metabolite separation, a BEH amide column (2.1 mm × 100 mm, 1.7 µm; Waters) was maintained at 40°C, and data acquisition was conducted in positive ion electrospray mode. Spectral peaks were automatically calibrated to correct for locked‐mass deviations. Data were scaled and normalized prior to principal component analysis (PCA) using R software. Differential metabolites were identified based on fold change thresholds and Bonferroni‐adjusted *p*‐values. Metabolite annotation was performed against BGI HR‐PMDB, mzCloud, HMDB, and KEGG databases using precursor ion, MS/MS spectra, and retention time information where applicable. Metabolites annotated with confidence Levels 1–4 were considered identified, as defined in Table S2. Pathway enrichment analysis was then performed to determine the biological relevance of the altered metabolites.

### 4.14 Fecal Metagenomic Sequencing and Functional Annotation

Fecal microbial DNA was extracted using the CTAB method, and metagenomic libraries were prepared using the Fast DNA Library Prep Set for Illumina. After DNA fragmentation, end repair, A‐tailing, adapter ligation, size selection, and PCR amplification, sequencing was performed by LC‐BIO Technologies (Hangzhou, China). Raw reads were processed by removing adapters with cutadapt v1.9, trimming low‐quality reads with fqtrim v0.94, and removing host‐derived reads against the mouse reference genome using Bowtie2 v2.2.0. Clean reads were *de novo* assembled using MEGAHIT v1.2.9. Coding sequences were predicted using MetaGeneMark v3.26 and clustered using CD‐HIT v4.6.1 to generate a non‐redundant unigene catalogue. Unigene abundance was estimated as transcripts per million based on read mapping with Bowtie2 v2.2.0.

Taxonomic annotation was performed against the NCBI NR database using DIAMOND v0.9.14 with the lowest common ancestor approach, and functional annotation was performed against KEGG, GO, eggNOG, CAZy, CARD, PHI, MGEs, and VFDB databases. Alpha diversity indices, including Chao, Shannon, and Simpson indices, were calculated, and PCA was used to visualize overall microbial compositional differences. Differentially enriched taxa were identified using LEfSe, with an LDA score threshold of >2.5 and *p* < 0.05. For functional contribution analysis, tryptophan metabolism‐related enzymes were extracted according to KEGG and EC annotations. IAH was defined as EC 3.5.1.4, and TnaA was defined as EC 4.1.99.1. Species‐level contributions to tryptophan metabolism, IAH abundance, and TnaA abundance were calculated by integrating taxonomic assignments with the abundance profiles of corresponding functional genes.

### 4.15 Stereotaxic Viral Injection and Retrograde Tracing

Mice were deeply anesthetized with 2% isoflurane and fixed in a stereotaxic apparatus. For neuronal AhR knockdown, saline‐ or VPA‐exposed male offspring received bilateral injection of pAAV‐hSyn‐shAhr or pAAV‐hSyn‐shNC into the retrosplenial agranular cortex (RSA; AP, −1.80 mm; ML, ±0.60 mm; DV, −0.70 mm relative to bregma). A total volume of 250 nL virus per side was delivered at a constant rate of 50 nL/min using a glass micropipette. The viral titer was 1.0 × 10^1^ ^3^ vg/mL (ObiO, Shanghai, China). After injection, the micropipette was left in place for 10 min to minimize backflow and then slowly withdrawn. The skull opening was sealed with bone wax, and the skin was sutured. Mice received daily intraperitoneal injection of cefazolin sodium (25 mg/kg) for 3 consecutive days after surgery.

For retrograde tracing, PRV‐EGFP was injected into the hippocampal CA3 region of C57BL/6J mice. The injection coordinates were AP, −1.80 mm; ML, ±2.40 mm; and DV, −2.20 mm relative to bregma. PRV‐EGFP (2.19 × 10^8^ pfu/mL; BrainVTA, Wuhan, China) was bilaterally injected at a volume of 100 nL per side at a constant rate of 100 nL/min. After injection, the micropipette was kept in place for 10 min before slow withdrawal. The skull opening was sealed with bone wax, and the skin was sutured. Brain tissues were collected at the indicated time points after PRV‐EGFP injection for fluorescence imaging of retrogradely labeled neurons.

### 4.16 Statistical Analysis

Data are presented as mean ± SEM unless otherwise stated. The sample size (n) represents the number of biological replicates, animals, independent cultures, or human participants, as indicated in the corresponding figure legends. No data were excluded from the analysis unless predefined quality‐control criteria were not met. For datasets requiring preprocessing, normalization or transformation was performed as described in the corresponding Methods sections.

Statistical analyses were performed using GraphPad Prism 10.6 (GraphPad Software Inc., San Diego, CA, USA). Data normality was assessed using the Shapiro‐Wilk test or Kolmogorov‐Smirnov test, and homogeneity of variance was evaluated using the Brown‐Forsythe test. For comparisons among multiple groups, one‐way ANOVA followed by Tukey's post hoc test was used for normally distributed data with equal variances. Welch's ANOVA followed by Games‐Howell post hoc test was used when the assumption of equal variance was not met. For non‐normally distributed data, the Kruskal‐Wallis test followed by Dunn's post hoc test was applied. For pairwise comparisons, paired or unpaired Student's t‐test was used as appropriate according to the experimental design. Specifically, paired Student's *t*‐test was used for Figure 2B,C,G–J; Figure 7B,C,E,F,K,L,N,O; Figure 8C,D, Figure S10D; and Figure S11D. Unpaired Student's *t*‐test was used for Figure 6C; Figure 7D,H,I,M,Q–T; Figure S10A–C, E–J; and Supplemental Figure S11A–C,E–H. Two‐way repeated‐measures ANOVA followed by Sidak's multiple comparisons test was used for time‐course fluorescence tracking data in Figure 1E,F. Correlation analyses were performed using Spearman's correlation analysis. Data visualization was performed using GraphPad Prism 10.6, Chiplot (https://www.chiplot.online, China), and Wei Sheng Xin (http://www.bioinformatics.com.cn/, China). Statistical significance was defined as *p* < 0.05. Unless otherwise indicated, significance levels are denoted as ^*^ *p* < 0.05, ^**^ *p* < 0.01, and ^***^ *p* < 0.001.

## Author Contributions

**Rui Guo**: Writing – original draft, Validation, Methodology, Investigation, Formal analysis, Data curation, Funding acquisition. **Caian He**: Writing – original draft, Methodology, Investigation, Formal analysis. **Xiao Xiao**: Investigation, Formal analysis, Conceptualization. **Jingmeng Li**: Methodology, Validation, Investigation. **Zilong Zhang**: Formal analysis, Visualization. **Ying He**: Data curation, Software. **Chuanchuan Wang**: Data curation, Visualization. **Jiale Zhao**: Methodology, Software. **Jun Gong**: Validation. **Jiarui Liang**: Supervision. **Tian Yuan**: Resources, Writing – review & editing. **Haiting Sun**: Collection of stool samples from the population. **Xuebo Liu** and **Chao Gao**: Supervision, Resources, Project administration, Funding acquisition. **Zhigang Liu**: Resources, Project administration, Funding acquisition.

## Ethics Statement

All animal procedures were approved by the Animal Ethics Committee of Northwest A&F University under approval no. XN2023‐0612. Human fecal sample collection for the in vitro fermentation assay was approved by the Medical Ethics Committee of the First Affiliated Hospital of Air Force Medical University under approval no. KY20242174‐F‐1. Written informed consent was obtained from the parents or legal guardians of all participating children.

## Conflicts of Interest

The authors declare no conflicts of interest.

## Supporting information




**Supporting File 1**: advs77222‐sup‐0001‐SuppMat.docx.

**Supporting File 2**: advs77222‐sup‐0002‐TableS1.xlsx.

**Supporting File 3**: advs77222‐sup‐0003‐TableS2.xlsx.

**Supporting File 4**: advs77222‐sup‐0004‐TableS3.xlsx.

**Supporting File 5**: advs77222‐sup‐0005‐TableS4.xlsx.

**Supporting File 6**: advs77222‐sup‐0006‐TableS5.xlsx.

**Supporting File 7**: advs77222‐sup‐0007‐TableS6.xlsx.

**Supporting File 8**: advs77222‐sup‐0008‐TableS7.xlsx.

**Supporting File 9**: advs77222‐sup‐0009‐TableS8.xlsx.

**Supporting File 10**: advs77222‐sup‐0010‐TableS9.xlsx.

**Supporting File 11**: advs77222‐sup‐0011‐TableS10.xlsx.

## Data Availability

Raw mouse fecal metagenomic sequencing data generated in this study have been deposited in GSA under accession CRA044199. Public human metagenomic and metabolomic datasets were obtained from Zhang et al. and Needham et al., respectively, as described in Methods. Raw microscopy images, Western blot files, quantitative source data, and other numerical data supporting the figures are provided as Supporting Information. Original figures have been deposited in Figshare data set as follows: https://figshare.com/s/1dbc6c95309fd66f3b24.
