# Supplementary material for: Targeted Restoration of the Microbial Tryptophan Oxidative Pathway Ameliorates Autism‐Like Behavioral and Synaptic Deficits
Source: Adv Sci (Weinh). 2026 Sep 27:e22265. Online ahead of print. doi: 10.1002/advs.202522265 (PMC13616266; doi:10.1002/advs.202522265)
Supplement: Supplementary file 1 [file ADVS-9999-e22265-s002.docx]

**Supplemental Figure 1 | Formulation and *in vitro* metabolic characterization of the targeted synbiotic.**

(A) Whole-genome analysis of Lacticaseibacillus rhamnosus C502 with genes encoding tryptophan-metabolizing enzymes (red-labeled in Figure 1A). (B) Growth curves of *L.R.* C502 in 10 g/L different carbon source medium (n = 5).

**Supplemental Figure 2 | Concentrations of tryptophan-related metabolites in fermentation supernatants after 48 h.**

(A) L-tryptophan, (B) indole-3-lactic acid (ILA), and (C) indole-3-propionic acid (IPA), respectively. ASD pool (n = 6 technical replicates) and TD pool (n = 4 technical replicates) were generated from five male ASD children and five male TD children, respectively.

**Supplemental Figure 3 | Social behaviors of VPA-induced mice with different interventions.** (A) Representative locomotor trajectories in the three-chamber social interaction test. (B-C) Interaction index for sociability and social novelty preference (n = 15). Data shown as mean ± SEM. Statistical analysis details are provided in Methods.

**Supplemental Figure 4 | Histological analysis of hippocampal CA3 area in mice with different interventions.** (A) Representative images of Hematoxylin and Eosin (H&E); (B) Nissl's staining of hippocampal CA3 area; (C) Quantification of neurons in hippocampal CA3 area (n = 4).

**Supplemental Figure 5 | KEGG enrichment analysis of differential metabolites between groups.**

(A) VPA and VPA+G groups; (B) VPA and VPA+L groups.

**Supplemental Figure 6 | Representative images of H&E** staining o**f colon sections.**

**Supplemental Figure 7 | Gut microbiota diversity and discriminative taxa after targeted interventions.**

(A) Alpha diversity indices; (B) Principal component analysis of microbial profiles; (C–D) LEfSe analysis of discriminative bacterial taxa between VPA and VPA+G or VPA+L groups.

**Supplemental Figure 8 | Metabolic characteristics of *L.R.* C502**

(A–C) Concentrations of IAA, IAM, and IAld after incubation with *L.R.* C502 under different IAM concentrations. (D–H) Concentrations of L-tryptophan, indole, tryptamine, ILA, and IPA after incubation. Metabolite concentrations were normalized to bacterial abundance. Data are shown as mean ± SEM. Statistical analysis was not performed because these assays were intended as exploratory metabolic characterization based on technical replicates.

**Supplemental Figure 9 | Spearman correlation analysis of tryptophan-related metabolites with gut-brain axis parameters.**

**Supplemental Figure 10 | Dependence of the targeted intervention strategy on tryptophan-metabolizing microbiota.**

(A-B) Interaction index for sociability and social novelty (n = 11). (C) Fiber photometry recordings of CA3 neuronal activity (n = 5). (D) Peak value of GCaMP6m signals (n = 5). (E-H) Quantification of number of synaptic vesicles, area ratio of pre- to post-synaptic terminals, PSD length, and PSD thickness (n = 3). (I) Serum levels of IAA, IPA, and ILA (n = 8). (J) Relative mRNA expression of *Shank3*, *Snap25*, and *Stx1a* in hippocampus (n = 8). Data shown as mean ± SEM. Statistical analysis details are provided in Methods.

**Supplemental Figure 11 | Effects of IAA administration in VPA-induced mice.**

(A-B) Interaction index for sociability and social novelty (n = 11). (C) Fiber photometry recordings of CA3 neuronal activity (n = 5). (D) Peak value of GCaMP6m signals (n = 5). (E-F) Quantification of number of area ratio of pre- to post-synaptic terminals, and PSD thickness (n = 3). (G) Serum levels of IAA, IPA, and ILA (n = 8). (H) Relative mRNA expression of *Shank3*, *Snap25*, and *Stx1a* in hippocampus (n = 8). Data shown as mean ± SEM. Statistical analysis details are provided in Methods.

**Supplemental Figure 12 | Supporting evidence and validation for RSA-targeted AhR knockdown.**

(A) Representative images of PRV-EGFP retrograde tracing from CA3 to the RSA/RSG region at 24, 48, and 72 h after injection. (B) Representative fluorescence images showing viral expression in the bilateral RSA regions. (C) Representative locomotor trajectories in the three-chamber social interaction test. (D–E) Interaction indices for sociability and social novelty (n = 10). (F–G) Relative mRNA expression of AhR downstream targets *Cyp1a1* and *Cyp1b1* in the hippocampus (n = 5). Data are shown as mean ± SEM. Statistical analysis details are provided in Methods.
